# On Relations Between the Relative Entropy and *χ*^2^-Divergence, Generalizations and Applications

**DOI:** 10.3390/e22050563

**Published:** 2020-05-18

**Authors:** Tomohiro Nishiyama, Igal Sason

**Affiliations:** 1 Independent Researcher, Tokyo 206–0003, Japan; htam0ybboh@gmail.com; 2 Faculty of Electrical Engineering, Technion—Israel Institute of Technology, Technion City, Haifa 3200003, Israel

**Keywords:** relative entropy, chi-squared divergence, *f*-divergences, method of types, large deviations, strong data–processing inequalities, information contraction, maximal correlation, Markov chains

## Abstract

This paper is focused on a study of integral relations between the relative entropy and the chi-squared divergence, which are two fundamental divergence measures in information theory and statistics, a study of the implications of these relations, their information-theoretic applications, and some generalizations pertaining to the rich class of *f*-divergences. Applications that are studied in this paper refer to lossless compression, the method of types and large deviations, strong data–processing inequalities, bounds on contraction coefficients and maximal correlation, and the convergence rate to stationarity of a type of discrete-time Markov chains.

## 1. Introduction

The relative entropy (also known as the Kullback–Leibler divergence [1]) and the chi-squared divergence [2] are divergence measures which play a key role in information theory, statistics, learning, signal processing, and other theoretical and applied branches of mathematics. These divergence measures are fundamental in problems pertaining to source and channel coding, combinatorics and large deviations theory, goodness-of-fit and independence tests in statistics, expectation–maximization iterative algorithms for estimating a distribution from an incomplete data, and other sorts of problems (the reader is referred to the tutorial paper by Csiszár and Shields [3]). They both belong to an important class of divergence measures, defined by means of convex functions *f*, and named *f*-divergences [4,5,6,7,8]. In addition to the relative entropy and the chi-squared divergence, this class unifies other useful divergence measures such as the total variation distance in functional analysis, and it is also closely related to the Rényi divergence which generalizes the relative entropy [9,10]. In general, *f*-divergences (defined in Section 2) are attractive since they satisfy pleasing features such as the data–processing inequality, convexity, (semi)continuity, and duality properties, and they therefore find nice applications in information theory and statistics (see, e.g., [6,8,11,12]).

In this work, we study integral relations between the relative entropy and the chi-squared divergence, implications of these relations, and some of their information-theoretic applications. Some generalizations which apply to the class of *f*-divergences are also explored in detail. In this context, it should be noted that integral representations of general *f*-divergences, expressed as a function of either the DeGroot statistical information [13], the $E_{\gamma }$-divergence (a parametric sub-class of *f*-divergences, which generalizes the total variation distance [14] [p. 2314]) and the relative information spectrum, have been derived in [12] [Section 5], [15] [Section 7.B], and [16] [Section 3], respectively.

Applications in this paper are related to lossless source compression, large deviations by the method of types, and strong data–processing inequalities. The relevant background for each of these applications is provided to make the presentation self contained.

We next outline the paper contributions and the structure of our manuscript.

### 1.1. Paper Contributions

This work starts by introducing integral relations between the relative entropy and the chi-squared divergence, and some inequalities which relate these two divergences (see Theorem 1, its corollaries, and Proposition 1). It continues with a study of the implications and generalizations of these relations, pertaining to the rich class of *f*-divergences. One implication leads to a tight lower bound on the relative entropy between a pair of probability measures, expressed as a function of the means and variances under these measures (see Theorem 2). A second implication of Theorem 1 leads to an upper bound on a skew divergence (see Theorem 3 and Corollary 3). Due to the concavity of the Shannon entropy, let the concavity deficit of the entropy function be defined as the non-negative difference between the entropy of a convex combination of distributions and the convex combination of the entropies of these distributions. Then, Corollary 4 provides an upper bound on this deficit, expressed as a function of the pairwise relative entropies between all pairs of distributions. Theorem 4 provides a generalization of Theorem 1 to the class of *f*-divergences. It recursively constructs non-increasing sequences of *f*-divergences and as a consequence of Theorem 4 followed by the usage of polylogairthms, Corollary 5 provides a generalization of the useful integral relation in Theorem 1 between the relative entropy and the chi-squared divergence. Theorem 5 relates probabilities of sets to *f*-divergences, generalizing a known and useful result by Csiszár for the relative entropy. With respect to Theorem 1, the integral relation between the relative entropy and the chi-squared divergence has been independently derived in [17], which also derived an alternative upper bound on the concavity deficit of the entropy as a function of total variational distances (differing from the bound in Corollary 4, which depends on pairwise relative entropies). The interested reader is referred to [17], with a preprint of the extended version in [18], and to [19] where the connections in Theorem 1 were originally discovered in the quantum setting.

The second part of this work studies information-theoretic applications of the above results. These are ordered by starting from the relatively simple applications, and ending at the more complicated ones. The first one includes a bound on the redundancy of the Shannon code for universal lossless compression with discrete memoryless sources, used in conjunction with Theorem 3 (see Section 4.1). An application of Theorem 2 in the context of the method of types and large deviations analysis is then studied in Section 4.2, providing non-asymptotic bounds which lead to a closed-form expression as a function of the Lambert *W*-function (see Proposition 2). Strong data–processing inequalities with bounds on contraction coefficients of skew divergences are provided in Theorem 6, Corollary 7 and Proposition 3. Consequently, non-asymptotic bounds on the convergence to stationarity of time-homogeneous, irreducible, and reversible discrete-time Markov chains with finite state spaces are obtained by relying on our bounds on the contraction coefficients of skew divergences (see Theorem 7). The exact asymptotic convergence rate is also obtained in Corollary 8. Finally, a property of maximal correlations is obtained in Proposition 4 as an application of our starting point on the integral relation between the relative entropy and the chi-squared divergence.

### 1.2. Paper Organization

This paper is structured as follows. Section 2 presents notation and preliminary material which is necessary for, or otherwise related to, the exposition of this work. Section 3 refers to the developed relations between divergences, and Section 4 studies information-theoretic applications. Proofs of the results in Section 3 and Section 4 (except for short proofs) are deferred to Section 5.

## 2. Preliminaries and Notation

This section provides definitions of divergence measures which are used in this paper, and it also provides relevant notation.

**Definition** **1.** *[12] [p. 4398] Let P and Q be probability measures, let μ be a dominating measure of P and Q (i.e., P,Q≪μ), and let $p:=\frac{dP}{d\mu }$ and $q:=\frac{dQ}{d\mu }$ be the densities of P and Q with respect to μ. The* *f*-divergence *from P to Q is given by*

(1) $$\begin{array}{c} D_{f}\left(P∥Q\right):=\int q\;f\left(\frac{p}{q}\right)\;d\mu , \end{array}$$

*where*

(2) $$\begin{array}{cc} \; & f\left(0\right):=\lim_{t\to 0^{+}}\;f\left(t\right),\;0f\left(\frac{0}{0}\right):=0, \end{array}$$

(3) $$\begin{array}{cc} \; & 0f\left(\frac{a}{0}\right):=\lim_{t\to 0^{+}}\;tf\left(\frac{a}{t}\right)=a\lim_{u\to \infty }\frac{f(u)}{u},\;a>0. \end{array}$$

*It should be noted that the right side of (1) does not depend on the dominating measure μ.*

Throughout the paper, we denote by 1{relation} the indicator function; it is equal to 1 if the relation is true, and it is equal to 0 otherwise. Throughout the paper, unless indicated explicitly, logarithms have an arbitrary common base (that is larger than 1), and exp(·) indicates the inverse function of the logarithm with that base.

**Definition** **2.** *[1] The relative entropy is the f-divergence with f(t):=tlogt for t>0,*

(4)D(P∥Q):=Df(P∥Q)(5)=∫plogpqdμ.

**Definition** **3.** *The total variation distance between probability measures P and Q is the f-divergence from P to Q with f(t):=|t−1| for all t≥0. It is a symmetric f-divergence, denoted by |P−Q|, which is given by*

(6)|P−Q|:=Df(P∥Q)(7)=∫|p−q|dμ.

**Definition** **4.** *[2] The chi-squared divergence from P to Q is defined to be the f-divergence in (1) with $f\left(t\right):={\left(t-1\right)}^{2}$ or $f\left(t\right):=t^{2}-1$ for all t>0,*

(8)χ2(P∥Q):=Df(P∥Q)(9)=∫(p−q)2qdμ=∫p2qdμ−1.

The Rényi divergence, a generalization of the relative entropy, was introduced by Rényi [10] in the special case of finite alphabets. Its general definition is given as follows (see, e.g., [9]).

**Definition** **5.** *[10] Let P and Q be probability measures on X dominated by μ, and let their densities be respectively denoted by $p=\frac{dP}{d\mu }$ and $q=\frac{dQ}{d\mu }$. The* Rényi divergence *of order α∈[0,∞] is defined as follows:*

- *If α∈(0,1)∪(1,∞), then*
  (10)Dα(P∥Q)=1α−1logEpα(Z)q1−α(Z)(11)=1α−1log∑x∈XPα(x)Q1−α(x),
  *where Z∼μ in (10), and (11) holds if X is a discrete set.*
- *By the continuous extension of $D_{\alpha }\left(P∥Q\right)$,*
  (12)D0(P∥Q)=maxA:P(A)=1log1Q(A),(13)D1(P∥Q)=D(P∥Q),(14)D∞(P∥Q)=logess supp(Z)q(Z).

The second-order Rényi divergence and the chi-squared divergence are related as follows:

(15) $$\begin{array}{c} D_{2}\left(P∥Q\right)=\log\left(1+\chi^{2}\left(P∥Q\right)\right), \end{array}$$

and the relative entropy and the chi-squared divergence satisfy (see, e.g., [20] [Theorem 5])

(16) $$\begin{array}{c} D\left(P∥Q\right)\leq \log\left(1+\chi^{2}\left(P∥Q\right)\right). \end{array}$$

Inequality (16) readily follows from (13), (15), and since $D_{\alpha }\left(P∥Q\right)$ is monotonically increasing in α∈(0,∞) (see [9] [Theorem 3]). A tightened version of (16), introducing an improved and locally-tight upper bound on D(P∥Q) as a function of $\chi^{2}\left(P∥Q\right)$ and $\chi^{2}\left(Q∥P\right)$, is introduced in [15] [Theorem 20]. Another sharpened version of (16) is derived in [15] [Theorem 11] under the assumption of a bounded relative information. Furthermore, under the latter assumption, tight upper and lower bounds on the ratio $\frac{D(P∥Q)}{\chi^{2}\left(P∥Q\right)}$ are obtained in [15] [(169)].

**Definition** **6.** *[21] The Györfi–Vajda divergence of order s∈[0,1] is an f-divergence with*

(17) $$\begin{array}{c} f\left(t\right)=\phi_{s}\left(t\right):=\frac{{\left(t-1\right)}^{2}}{s+(1-s)t},\;t\geq 0. \end{array}$$

*Vincze–Le Cam distance (also known as the triangular discrimination) ([22,23]) is a special case with $s=\frac{1}{2}$.*

In view of (1), (9) and (17), it can be verified that the Györfi–Vajda divergence is related to the chi-squared divergence as follows:

(18) $$D_{\phi_{s}}\left(P∥Q\right)=\{\begin{array}{ll} \frac{1}{s^{2}}\cdot \chi^{2}\left(P\;∥\;(1-s)P+sQ\right),\; & \;s\in \left(0,1\right],\\ \chi^{2}\left(Q∥P\right), & \;s=0. \end{array}$$

Hence,

(19)Dϕ1(P∥Q)=χ2(P∥Q),(20)Dϕ0(P∥Q)=χ2(Q∥P).

## 3. Relations between Divergences

We introduce in this section results on the relations between the relative entropy and the chi-squared divergence, their implications, and generalizations. Information–theoretic applications are studied in the next section.

### 3.1. Relations between the Relative Entropy and the Chi-Squared Divergence

The following result relates the relative entropy and the chi-squared divergence, which are two fundamental divergence measures in information theory and statistics. This result was recently obtained in an equivalent form in [17] [(12)] (it is noted that this identity was also independently derived by the coauthors in two separate un-published works in [24] [(16)] and [25]). It should be noted that these connections between divergences in the quantum setting were originally discovered in [19] [Theorem 6]. Beyond serving as an interesting relation between these two fundamental divergence measures, it is introduced here for the following reasons:

(a) New consequences and applications of it are obtained, including new shorter proofs of some known results;
(b) An interesting extension provides new relations between *f*-divergences (see Section 3.3).

**Theorem** **1.** *Let P and Q be probability measures defined on a measurable space (X,F), and let*

(21) $$\begin{array}{c} R_{\lambda }:=\left(1-\lambda \right)P+\lambda Q,\;\lambda \in \left[0,1\right] \end{array}$$

*be the convex combination of P and Q. Then, for all λ∈[0,1],*

(22) $$\begin{array}{cc} \frac{1}{\log e}\;D\left(P∥R_{\lambda }\right)\; & =\int_{0}^{\lambda }\chi^{2}\left(P∥R_{s}\right)\;\frac{ds}{s}, \end{array}$$

(23) $$\begin{array}{cc} \frac{1}{2}\;\lambda^{2}\;\chi^{2}\left(R_{1-\lambda }∥Q\right)\; & =\int_{0}^{\lambda }\chi^{2}\left(R_{1-s}∥Q\right)\;\frac{ds}{s}. \end{array}$$

**Proof.**  See Section 5.1. □

A specialization of Theorem 1 by letting λ=1 gives the following identities.

**Corollary** **1.** (24) $$\begin{array}{c} \frac{1}{\log e}\;D\left(P∥Q\right)=\int_{0}^{1}\chi^{2}\left(P\;∥\;\left(1-s\right)P+sQ\right)\;\frac{ds}{s}, \end{array}$$

(25) $$\begin{array}{c} \frac{1}{2}\;\chi^{2}\left(P∥Q\right)=\int_{0}^{1}\chi^{2}\left(sP+\left(1-s\right)Q\;∥\;Q\right)\;\frac{ds}{s}. \end{array}$$

**Remark** **1.** *The substitution $s:=\frac{1}{1+t}$ transforms (24) to [26] [Equation (31)], i.e.,*

(26) $$\begin{array}{c} \frac{1}{\log e}\;D\left(P∥Q\right)=\int_{0}^{\infty }\chi^{2}\left(P\;∥\;\frac{tP+Q}{1+t}\right)\;\frac{dt}{1+t}. \end{array}$$

In view of (18) and (21), an equivalent form of (22) and (24) is given as follows:

**Corollary** **2.** *For s∈[0,1], let $\phi_{s}:\left[0,\infty \right)\to R$ be given in (17). Then,*

(27)1logeD(P∥Rλ)=∫0λsDϕs(P∥Q)ds,λ∈[0,1],(28)1logeD(P∥Q)=∫01sDϕs(P∥Q)ds.

By Corollary 1, we obtain original and simple proofs of new and old *f*-divergence inequalities.

**Proposition** **1.** (*f*-divergence inequalities).

*(a)*  *Pinsker’s inequality:*
  (29) $$\begin{array}{c} D\left(P∥Q\right)\geq \frac{1}{2}{\left|P-Q\right|}^{2}\log e. \end{array}$$
*(b)*  (30) $$\begin{array}{c} \frac{1}{\log e}\;D\left(P∥Q\right)\leq \frac{1}{3}\;\chi^{2}\left(P∥Q\right)+\frac{1}{6}\;\chi^{2}\left(Q∥P\right). \end{array}$$
  *Furthermore, let $\left\{P_{n}\right\}$ be a sequence of probability measures that is defined on a measurable space (X,F), and which converges to a probability measure P in the sense that*
  (31) $$\begin{array}{c} \lim_{n\to \infty }ess sup\frac{dP_{n}}{dP}\;\left(X\right)=1, \end{array}$$
  *with X∼P. Then, (30) is locally tight in the sense that its both sides converge to 0, and*
  (32) $$\begin{array}{c} \lim_{n\to \infty }\frac{\frac{1}{3}\;\chi^{2}\left(P_{n}∥P\right)+\frac{1}{6}\;\chi^{2}\left(P∥P_{n}\right)}{\frac{1}{\log e}\;D\left(P_{n}∥P\right)}=1. \end{array}$$
*(c)*  *For all θ∈(0,1),*
  (33) $$\begin{array}{c} D\left(P∥Q\right)\geq \left(1-\theta \right)\;\log\left(\frac{1}{1-\theta }\right)\;D_{\phi_{\theta }}\left(P∥Q\right). \end{array}$$
  *Moreover, under the assumption in (31), for all θ∈[0,1]*
  (34) $$\begin{array}{c} \lim_{n\to \infty }\frac{D(P∥P_{n})}{D_{\phi_{\theta }}\left(P∥P_{n}\right)}=\frac{1}{2}\log e. \end{array}$$
*(d)*  *[15] [Theorem 2]:*
  (35) $$\begin{array}{c} \frac{1}{\log e}\;D\left(P∥Q\right)\leq \frac{1}{2}\;\chi^{2}\left(P∥Q\right)+\frac{1}{4}\;\left|P-Q\right|. \end{array}$$

**Proof.**  See Section 5.2. □

**Remark** **2.** *Inequality (30) is locally tight in the sense that (31) yields (32). This property, however, is not satisfied by (16) since the assumption in (31) implies that*

(36) $$\begin{array}{c} \lim_{n\to \infty }\frac{\log\left(1+\chi^{2}\left(P_{n}∥P\right)\right)}{D(P_{n}∥P)}=2. \end{array}$$

**Remark** **3.** *Inequality (30) readily yields*

(37) $$\begin{array}{cc} \; & D\left(P∥Q\right)+D\left(Q∥P\right)\leq \frac{1}{2}\left(\chi^{2}\left(P∥Q\right)+\chi^{2}\left(Q∥P\right)\right)\;\log e, \end{array}$$

*which is proved by a different approach in [27] [Proposition 4]. It is further shown in [15] [Theorem 2 b)] that*

(38) $$\begin{array}{c} \sup\frac{D(P∥Q)+D(Q∥P)}{\chi^{2}\left(P∥Q\right)+\chi^{2}\left(Q∥P\right)}=\frac{1}{2}\log e, \end{array}$$

*where the supremum is over P≪≫Q and P≠Q.*

### 3.2. Implications of Theorem 1

We next provide two implications of Theorem 1. The first implication, which relies on the Hammersley–Chapman–Robbins (HCR) bound for the chi-squared divergence [28,29], gives the following tight lower bound on the relative entropy D(P∥Q) as a function of the means and variances under *P* and *Q*.

**Theorem** **2.** *Let P and Q be probability measures defined on the measurable space (R,B), where R is the real line and B is the Borel σ–algebra of subsets of R. Let $m_{P}$, $m_{Q}$, $\sigma_{P}^{2}$, and $\sigma_{Q}^{2}$ denote the expected values and variances of X∼P and Y∼Q, i.e.,*

(39) $$\begin{array}{cc} \; & E\left[X\right]=:m_{P},\;E\left[Y\right]=:m_{Q},\;\mathrm{Var}\left(X\right)=:\sigma_{P}^{2},\;\mathrm{Var}\left(Y\right)=:\sigma_{Q}^{2}. \end{array}$$

*(a)*  *If $m_{P}\neq m_{Q}$, then*
  (40) $$\begin{array}{c} D(P∥Q)\geq d(r∥s), \end{array}$$
  *where $d\left(r∥s\right):=r\log\frac{r}{s}+\left(1-r\right)\log\frac{1-r}{1-s}$, for r,s∈[0,1], denotes the binary relative entropy (with the convention that $0\log\frac{0}{0}=0$), and*
  (41)r:=12+b4av∈[0,1],(42)s:=r−a2v∈[0,1],(43)a:=mP−mQ,(44)b:=a2+σQ2−σP2,(45)v:=σP2+b24a2.
*(b)*  *The lower bound on the right side of (40) is attained for P and Q which are defined on the two-element set $U:=\{u_{1},u_{2}\}$, and*
  (46) $$\begin{array}{c} P\left(u_{1}\right)=r,\;Q\left(u_{1}\right)=s, \end{array}$$
  *with r and s in (41) and (42), respectively, and for $m_{P}\neq m_{Q}$*
  (47) $$\begin{array}{cc} \; & u_{1}:=m_{P}+\sqrt{\frac{\left(1-r\right)\sigma_{P}^{2}}{r}},\;u_{2}:=m_{P}-\sqrt{\frac{r\sigma_{P}^{2}}{1-r}}. \end{array}$$
*(c)*  *If $m_{P}=m_{Q}$ and $\sigma_{P}$ and $\sigma_{Q}$ are selected arbitrarily, then*
  (48) $$\begin{array}{c} \underset{P,Q}{\inf}\;D\left(P∥Q\right)=0, \end{array}$$
  *where the infimum on the left side of (48) is taken over all P and Q which satisfy (39).*

**Proof.**  See Section 5.3. □

**Remark** **4.** *Consider the case of the non-equal means in Items (a) and (b) of Theorem 2. If these means are fixed, then the infimum of D(P∥Q) is zero by choosing arbitrarily large equal variances. Suppose now that the non-equal means $m_{P}$ and $m_{Q}$ are fixed, as well as one of the variances (either $\sigma_{P}^{2}$ or $\sigma_{Q}^{2}$). Numerical experimentation shows that, in this case, the achievable lower bound in (40) is monotonically decreasing as a function of the other variance, and it tends to zero as we let the free variance tend to infinity. This asymptotic convergence to zero can be justified by assuming, for example, that $m_{P},m_{Q}$, and $\sigma_{Q}^{2}$ are fixed, and $m_{P}>m_{Q}$ (the other cases can be justified in a similar way). Then, it can be verified from (41)–(45) that*

(49) $$\begin{array}{c} r=\frac{{\left(m_{P}-m_{Q}\right)}^{2}}{\sigma_{P}^{2}}+O\left(\frac{1}{\sigma_{P}^{4}}\right),\;s=O\left(\frac{1}{\sigma_{P}^{4}}\right), \end{array}$$

*which implies that d(r∥s)→0 as we let $\sigma_{P}\to \infty$. The infimum of the relative entropy D(P∥Q) is therefore equal to zero since the probability measures P and Q in (46) and (47), which are defined on a two-element set and attain the lower bound on the relative entropy under the constraints in (39), have a vanishing relative entropy in this asymptotic case.*

**Remark** **5.** *The proof of Item (c) in Theorem 2 suggests explicit constructions of sequences of pairs probability measures $\left\{\left(P_{n},Q_{n}\right)\right\}$ such that*

*(a)*  *The means under $P_{n}$ and $Q_{n}$ are both equal to m (independently of n);*
*(b)*  *The variance under $P_{n}$ is equal to $\sigma_{P}^{2}$, and the variance under $Q_{n}$ is equal to $\sigma_{Q}^{2}$ (independently of n);*
*(c)*  *The relative entropy $D(P_{n}∥Q_{n})$ vanishes as we let n→∞.*

*This yields in particular (48).*

A second consequence of Theorem 1 gives the following result. Its first part holds due to the concavity of $\exp\left(-D(P∥\cdot )\right)$ (see [30] [Problem 4.2]). The second part is new, and its proof relies on Theorem 1. As an educational note, we provide an alternative proof of the first part by relying on Theorem 1.

**Theorem** **3.** *Let P≪Q, and F:[0,1]→[0,∞) be given by*

(50) $$\begin{array}{c} F\left(\lambda \right):=D\left(P\;∥\;(1-\lambda )P+\lambda Q\right),\;\forall \;\lambda \in \left[0,1\right]. \end{array}$$

*Then, for all λ∈[0,1],*

(51) $$\begin{array}{c} F\left(\lambda \right)\leq \log\left(\frac{1}{1-\lambda +\lambda \exp\left(-D(P∥Q)\right)}\right), \end{array}$$

*with an equality if λ=0 or λ=1. Moreover, F is monotonically increasing, differentiable, and it satisfies*

(52) $$\begin{array}{cc} \; & F^{\prime }\left(\lambda \right)\geq \frac{1}{\lambda }\left[\exp\left(F(\lambda )\right)-1\right]\log e,\;\forall \;\lambda \in \left(0,1\right], \end{array}$$

(53) $$\begin{array}{cc} \; & \lim_{\lambda \to 0^{+}}\frac{F^{\prime }\left(\lambda \right)}{\lambda }=\chi^{2}\left(Q∥P\right)\;\log e, \end{array}$$

*so the limit in (53) is twice as large as the value of the lower bound on this limit as it follows from the right side of (52).*

**Proof.**  See Section 5.4. □

**Remark** **6.** *By the convexity of the relative entropy, it follows that F(λ)≤λD(P∥Q) for all λ∈[0,1]. It can be verified, however, that the inequality 1−λ+λexp(−x)≥exp(−λx) holds for all x≥0 and λ∈[0,1]. Letting x:=D(P∥Q) implies that the upper bound on F(λ) on the right side of (51) is tighter than or equal to the upper bound λD(P∥Q) (with an equality if and only if either λ∈{0,1} or P≡Q).*

**Corollary** **3.** *Let ${\left\{P_{j}\right\}}_{j=1}^{m}$, with m∈N, be probability measures defined on a measurable space (X,F), and let ${\left\{\alpha_{j}\right\}}_{j=1}^{m}$ be a sequence of non-negative numbers that sum to 1. Then, for all i∈{1,…,m},*

(54) $$\begin{array}{cc} D\left(P_{i}\;∥\;\sum_{j=1}^{m}\alpha_{j}P_{j}\right) & \leq -\log\left(\alpha_{i}+\left(1-\alpha_{i}\right)\exp\left(-\frac{1}{1-\alpha_{i}}\sum_{j\neq i}\alpha_{j}\;D\left(P_{i}∥P_{j}\right)\right)\right). \end{array}$$

**Proof.**  For an arbitrary i∈{1,…,m}, apply the upper bound on the right side of (51) with $\lambda :=1-\alpha_{i}$, $P:=P_{i}$ and $Q:=\frac{1}{1-\alpha_{i}}\;\sum_{j\neq i}\alpha_{j}P_{j}$. The right side of (54) is obtained from (51) by invoking the convexity of the relative entropy, which gives $D\left(P_{i}∥Q\right)\leq \frac{1}{1-\alpha_{i}}\sum_{j\neq i}\alpha_{j}D\left(P_{i}∥P_{j}\right)$. □

The next result provides an upper bound on the non-negative difference between the entropy of a convex combination of distributions and the respective convex combination of the individual entropies (it is also termed as the concavity deficit of the entropy function in [17] [Section 3]).

**Corollary** **4.** *Let ${\left\{P_{j}\right\}}_{j=1}^{m}$, with m∈N, be probability measures defined on a measurable space (X,F), and let ${\left\{\alpha_{j}\right\}}_{j=1}^{m}$ be a sequence of non-negative numbers that sum to 1. Then,*

(55) $$\begin{array}{c} 0\leq H\left(\sum_{j=1}^{m}\alpha_{j}P_{j}\right)-\sum_{j=1}^{m}\alpha_{j}H\left(P_{j}\right)\leq -\sum_{i=1}^{m}\alpha_{i}\log\left(\alpha_{i}+\left(1-\alpha_{i}\right)\exp\left(-\frac{1}{1-\alpha_{i}}\sum_{j\neq i}\alpha_{j}\;D\left(P_{i}∥P_{j}\right)\right)\right). \end{array}$$

**Proof.**  The lower bound holds due to the concavity of the entropy function. The upper bound readily follows from Corollary 3, and the identity

(56) $$\begin{array}{c} H\left(\sum_{j=1}^{m}\alpha_{j}P_{j}\right)-\sum_{j=1}^{m}\alpha_{j}H\left(P_{j}\right)=\sum_{i=1}^{m}\alpha_{i}D\left(P_{i}\;∥\;\sum_{j=1}^{m}\alpha_{j}P_{j}\right). \end{array}$$

 □

**Remark** **7.** *The upper bound in (55) refines the known bound (see, e.g., [31] [Lemma 2.2])*

(57) $$\begin{array}{c} H\left(\sum_{j=1}^{m}\alpha_{j}P_{j}\right)-\sum_{j=1}^{m}\alpha_{j}H\left(P_{j}\right)\leq \sum_{j=1}^{m}\alpha_{j}\;\log\frac{1}{\alpha_{j}}=H\left(\underline{\alpha }\right), \end{array}$$

*by relying on all the $\frac{1}{2}m\left(m-1\right)$ pairwise relative entropies between the individual distributions ${\left\{P_{j}\right\}}_{j=1}^{m}$. Another refinement of (57), expressed in terms of total variation distances, has been recently provided in [17] [Theorem 3.1].*

### 3.3. Monotonic Sequences of f-Divergences and an Extension of Theorem 1

The present subsection generalizes Theorem 1, and it also provides relations between *f*-divergences which are defined in a recursive way.

**Theorem** **4.** *Let P and Q be probability measures defined on a measurable space (X,F). Let $R_{\lambda }$, for λ∈[0,1], be the convex combination of P and Q as in (21). Let $f_{0}:\left(0,\infty \right)\to R$ be a convex function with $f_{0}\left(1\right)=0$, and let ${\left\{f_{k}\left(\cdot \right)\right\}}_{k=0}^{\infty }$ be a sequence of functions that are defined on (0,∞) by the recursive equation*

(58) $$\begin{array}{c} f_{k+1}\left(x\right):=\int_{0}^{1-x}f_{k}\left(1-s\right)\;\frac{ds}{s},\;x>0,\;\;k\in \left\{0,1,\ldots \right\}. \end{array}$$

*Then,*

*(a)*  *${\left\{D_{f_{k}}\left(P∥Q\right)\right\}}_{k=0}^{\infty }$ is a non-increasing (and non-negative) sequence of f-divergences.*
*(b)*  *For all λ∈[0,1] and k∈{0,1,…},*
  (59) $$\begin{array}{c} D_{f_{k+1}}\left(R_{\lambda }∥P\right)=\int_{0}^{\lambda }D_{f_{k}}\left(R_{s}∥P\right)\;\frac{ds}{s}. \end{array}$$

**Proof.**  See Section 5.5. □

We next use the polylogarithm functions, which satisfy the recursive equation [32] [Equation (7.2)]:

(60) $${\mathrm{Li}}_{k}\left(x\right):=\{\begin{array}{ll} \frac{x}{1-x}, & if\;k=0,\\ \int_{0}^{x}\frac{{\mathrm{Li}}_{k-1}\left(s\right)}{s}\;ds, & if\;k\geq 1. \end{array}$$

This gives ${\mathrm{Li}}_{1}\left(x\right)=-\log_{e}\left(1-x\right)$, ${\mathrm{Li}}_{2}\left(x\right)=-\int_{0}^{x}\frac{1}{s}\;\log_{e}\left(1-s\right)\;ds$ and so on, which are real–valued and finite for x<1.

**Corollary** **5.** *Let*

(61) $$\begin{array}{c} f_{k}\left(x\right):={\mathrm{Li}}_{k}\left(1-x\right),\;x>0,\;\;k\in \left\{0,1,\ldots \right\}. \end{array}$$

*Then, (59) holds for all λ∈[0,1] and k∈{0,1,…}. Furthermore, setting k=0 in (59) yields (22) as a special case.*

**Proof.**  See Section 5.6. □

### 3.4. On Probabilities and f-Divergences

The following result relates probabilities of sets to *f*-divergences.

**Theorem** **5.** *Let (X,F,μ) be a probability space, and let C∈F be a measurable set with μ(C)>0. Define the conditional probability measure*

(62) $$\begin{array}{c} \mu_{C}\left(E\right):=\frac{\mu (C\cap E)}{\mu (C)},\;\forall \;E\in F. \end{array}$$

*Let f:(0,∞)→R be an arbitrary convex function with f(1)=0, and assume (by continuous extension of f at zero) that $f\left(0\right):=\lim_{t\to 0^{+}}f\left(t\right)<\infty$. Furthermore, let $\tilde{f}:\left(0,\infty \right)\to R$ be the convex function which is given by*

(63) $$\begin{array}{c} \tilde{f}\left(t\right):=tf\left(\frac{1}{t}\right),\;\forall \;t>0. \end{array}$$

*Then,*

(64) $$\begin{array}{c} D_{f}\left(\mu_{C}∥\mu \right)=\tilde{f}\left(\mu (C)\right)+\left(1-\mu (C)\right)\;f\left(0\right). \end{array}$$

**Proof.**  See Section 5.7. □

Connections of probabilities to the relative entropy, and to the chi-squared divergence, are next exemplified as special cases of Theorem 5.

**Corollary** **6.** *In the setting of Theorem 5,*

(65) $$\begin{array}{cc} \; & D\left(\mu_{C}∥\mu \right)=\log\frac{1}{\mu \left(C\right)}, \end{array}$$

(66) $$\begin{array}{cc} \; & \chi^{2}\left(\mu_{C}∥\mu \right)=\frac{1}{\mu \left(C\right)}-1, \end{array}$$

*so (16) is satisfied in this case with equality. More generally, for all α∈(0,∞),*

(67) $$\begin{array}{c} D_{\alpha }\left(\mu_{C}∥\mu \right)=\log\frac{1}{\mu \left(C\right)}. \end{array}$$

**Proof.**  See Section 5.7. □

**Remark** **8.** *In spite of its simplicity, (65) proved very useful in the seminal work by Marton on transportation–cost inequalities, proving concentration of measures by information-theoretic tools [33,34] (see also [35] [Chapter 8] and [36] [Chapter 3]). As a side note, the simple identity (65) was apparently first explicitly used by Csiszár (see [37] [Equation (4.13)]).*

## 4. Applications

This section provides applications of our results in Section 3. These include universal lossless compression, method of types and large deviations, and strong data–processing inequalities (SDPIs).

### 4.1. Application of Corollary 3: Shannon Code for Universal Lossless Compression

Consider m>1 discrete, memoryless, and stationary sources with probability mass functions ${\left\{P_{i}\right\}}_{i=1}^{m}$, and assume that the symbols are emitted by one of these sources with an a priori probability $\alpha_{i}$ for source no. *i*, where ${\left\{\alpha_{i}\right\}}_{i=1}^{m}$ are positive and sum to 1.

For lossless data compression by a universal source code, suppose that a single source code is designed with respect to the average probability mass function $P:=\sum_{j=1}^{m}\alpha_{j}P_{j}$.

Assume that the designer uses a Shannon code, where the code assignment for a symbol x∈X is of length $\ell \left(x\right)=\lceil \log\frac{1}{P(x)}\rceil$ bits (logarithms are on base 2). Due to the mismatch in the source distribution, the average codeword length $\ell_{\mathrm{avg}}$ satisfies (see [38] [Proposition 3.B])

(68) $$\begin{array}{c} \sum_{i=1}^{m}\alpha_{i}H\left(P_{i}\right)+\sum_{i=1}^{m}\alpha_{i}D\left(P_{i}∥P\right)\leq \ell_{\mathrm{avg}}\leq \sum_{i=1}^{m}\alpha_{i}H\left(P_{i}\right)+\sum_{i=1}^{m}\alpha_{i}D\left(P_{i}∥P\right)+1. \end{array}$$

The fractional penalty in the average codeword length, denoted by ν, is defined to be equal to the ratio of the penalty in the average codeword length as a result of the source mismatch, and the average codeword length in case of a perfect matching. From (68), it follows that

(69) $$\begin{array}{c} \frac{\sum_{j=1}^{m}\alpha_{i}\;D\left(P_{i}∥P\right)}{1+\sum_{j=1}^{m}\alpha_{i}H\left(P_{i}\right)}\leq \nu \leq \frac{1+\sum_{j=1}^{m}\alpha_{i}\;D\left(P_{i}∥P\right)}{\sum_{j=1}^{m}\alpha_{i}H\left(P_{i}\right)}. \end{array}$$

We next rely on Corollary 3 to obtain an upper bound on ν which is expressed as a function of the m(m−1) relative entropies $D(P_{i}∥P_{j})$ for all i≠j in {1,…,m}. This is useful if, e.g., the *m* relative entropies on the left and right sides of (69) do not admit closed form expressions, in contrast to the m(m−1) relative entropies $D(P_{i}∥P_{j})$ for i≠j. We next exemplify this case.

For i∈{1,…,m}, let $P_{i}$ be a Poisson distribution with parameter $\lambda_{i}>0$. For all i,j∈{1,…,m}, the relative entropy from $P_{i}$ to $P_{j}$ admits the closed-form expression

(70) $$\begin{array}{cc} \; & D\left(P_{i}∥P_{j}\right)=\lambda_{i}\;\log\left(\frac{\lambda_{i}}{\lambda_{j}}\right)+\left(\lambda_{j}-\lambda_{i}\right)\log e. \end{array}$$

From (54) and (70), it follows that

(71) $$\begin{array}{cc} D(P_{i}∥P) & \leq -\log\left(\alpha_{i}+\left(1-\alpha_{i}\right)\exp\left(-\frac{f_{i}\left(\underline{\alpha },\underline{\lambda }\right)}{1-\alpha_{i}}\right)\right), \end{array}$$

where

(72)fi(α_,λ_):=∑j≠iαjD(Pi∥Pj)(73)=∑j≠iαjλilogλiλj+(λj−λi)loge.

The entropy of a Poisson distribution, with parameter $\lambda_{i}$, is given by the integral representation [39,40,41]

(74) $$\begin{array}{c} H\left(P_{i}\right)=\lambda_{i}\log\left(\frac{e}{\lambda_{i}}\right)+\int_{0}^{\infty }\left(\lambda_{i}-\frac{1-e^{-\lambda_{i}\left(1-e^{-u}\right)}}{1-e^{-u}}\right)\frac{e^{-u}}{u}\;du\;\log e. \end{array}$$

Combining (69), (71) and (74) finally gives an upper bound on ν in the considered setup.

**Example** **1.** *Consider five discrete memoryless sources where the probability mass function of source no. i is given by $P_{i}=\mathrm{Poisson}\left(\lambda_{i}\right)$ with $\underline{\lambda }=\left[16,20,24,28,32\right]$. Suppose that the symbols are emitted from one of the sources with equal probability, so $\underline{\alpha }=\left[\frac{1}{5},\frac{1}{5},\frac{1}{5},\frac{1}{5},\frac{1}{5}\right]$. Let $P:=\frac{1}{5}\left(P_{1}+\ldots +P_{5}\right)$ be the average probability mass function of the five sources. The term $\sum_{i}\alpha_{i}\;D\left(P_{i}∥P\right)$, which appears in the numerators of the upper and lower bounds on ν (see (69)), does not lend itself to a closed-form expression, and it is not even an easy task to calculate it numerically due to the need to compute an infinite series which involves factorials. We therefore apply the closed-form upper bound in (71) to get that $\sum_{i}\alpha_{i}\;D\left(P_{i}∥P\right)\leq 1.46$ bits, whereas the upper bound which follows from the convexity of the relative entropy (i.e., $\sum_{i}\alpha_{i}f_{i}\left(\underline{\alpha },\underline{\lambda }\right)$) is equal to 1.99 bits (both upper bounds are smaller than the trivial bound $\log_{2}5\approx 2.32$ bits). From (69), (74), and the stronger upper bound on $\sum_{i}\alpha_{i}\;D\left(P_{i}∥P\right)$, the improved upper bound on ν is equal to 57.0% (as compared to a looser upper bound of 69.3%, which follows from (69), (74), and the looser upper bound on $\sum_{i}\alpha_{i}\;D\left(P_{i}∥P\right)$ that is equal to 1.99 bits).*

### 4.2. Application of Theorem 2 in the Context of the Method of Types and Large Deviations Theory

Let $X^{n}=\left(X_{1},\ldots ,X_{n}\right)$ be a sequence of i.i.d. random variables with $X_{1}∼Q$, where *Q* is a probability measure defined on a finite set X, and Q(x)>0 for all x∈X. Let P be a set of probability measures on X such that Q∉P, and suppose that the closure of P coincides with the closure of its interior. Then, by Sanov’s theorem (see, e.g., [42] [Theorem 11.4.1] and [43] [Theorem 3.3]), the probability that the empirical distribution ${\hat{P}}_{X^{n}}$ belongs to P vanishes exponentially at the rate

(75) $$\begin{array}{c} \lim_{n\to \infty }\frac{1}{n}\;\log\frac{1}{P[{\hat{P}}_{X^{n}}\in P]}=\underset{P\in P}{\inf}D\left(P∥Q\right). \end{array}$$

Furthermore, for finite *n*, the method of types yields the following upper bound on this rare event:

(76)P[P^Xn∈P]≤n+|X|−1|X|−1exp−ninfP∈PD(P∥Q)(77)≤(n+1)|X|−1exp−ninfP∈PD(P∥Q),

whose exponential decay rate coincides with the exact asymptotic result in (75).

Suppose that *Q* is not fully known, but its mean $m_{Q}$ and variance $\sigma_{Q}^{2}$ are available. Let $m_{1}\in R$ and $\delta_{1},\varepsilon_{1},\sigma_{1}>0$ be fixed, and let P be the set of all probability measures *P*, defined on the finite set X, with mean $m_{P}\in \left[m_{1}-\delta_{1},m_{1}+\delta_{1}\right]$ and variance $\sigma_{P}^{2}\in \left[\sigma_{1}^{2}-\varepsilon_{1},\sigma_{1}^{2}+\varepsilon_{1}\right]$, where $|m_{1}-m_{Q}|>\delta_{1}$. Hence, P coincides with the closure of its interior, and Q∉P.

The lower bound on the relative entropy in Theorem 2, used in conjunction with the upper bound in (77), can serve to obtain an upper bound on the probability of the event that the empirical distribution of $X^{n}$ belongs to the set P, regardless of the uncertainty in *Q*. This gives

(78) $$\begin{array}{cc} P[{\hat{P}}_{X^{n}}\in P] & \leq {\left(n+1\right)}^{|X|-1}\;\exp\left(-nd^{*}\right), \end{array}$$

where

(79) $$\begin{array}{c} d^{*}:=\underset{m_{P},\sigma_{P}^{2}}{\inf}d\left(r∥s\right), \end{array}$$

and, for fixed $\left(m_{P},m_{Q},\sigma_{P}^{2},\sigma_{Q}^{2}\right)$, the parameters *r* and *s* are given in (41) and (42), respectively.

Standard algebraic manipulations that rely on (78) lead to the following result, which is expressed as a function of the Lambert–W function [44]. This function, which finds applications in various engineering and scientific fields, is a standard built–in function in mathematical software tools such as Mathematica, Matlab, and Maple. Applications of the Lambert–W function in information theory and coding are briefly surveyed in [45].

**Proposition** **2.** *For ε∈(0,1), let $n^{*}:=n^{*}\left(\varepsilon \right)$ denote the minimal value of n∈N such that the upper bound on the right side of (78) does not exceed ε∈(0,1). Then, $n^{*}$ admits the following closed-form expression:*

(80) $$\begin{array}{c} n^{*}=\max\left\{\left\lceil -\frac{\left(|X|-1\right)\;W_{-1}\left(\eta \right)\;\log e}{d^{*}}\right\rceil -1,\;1\right\}, \end{array}$$

*with*

(81) $$\begin{array}{cc} \; & \eta :=-\;\frac{d^{*}\;{\left(\varepsilon \;\exp(-d^{*})\right)}^{1/(|X|-1)}}{\left(|X|-1\right)\log e}\in [-\frac{1}{e},0), \end{array}$$

*and $W_{-1}\left(\cdot \right)$ on the right side of (80) denotes the secondary real–valued branch of the Lambert–W function (i.e., $x:=W_{-1}\left(y\right)$ where $W_{-1}:[-\frac{1}{e},0)\to \left(-\infty ,-1\right]$ is the inverse function of $y:=xe^{x}$).*

**Example** **2.** *Let Q be an arbitrary probability measure, defined on a finite set X, with mean $m_{Q}=40$ and variance $\sigma_{Q}^{2}=20$. Let P be the set of all probability measures P, defined on X, whose mean $m_{P}$ and variance $\sigma_{P}^{2}$ lie in the intervals [43,47] and [18,22], respectively. Suppose that it is required that, for all probability measures Q as above, the probability that the empirical distribution of the i.i.d. sequence $X^{n}∼Q^{n}$ that is included in the set P is at most $\varepsilon =10^{-10}$. We rely here on the upper bound in (78), and impose the stronger condition where it should not exceed ε. By this approach, it is obtained numerically from (79) that $d^{*}=0.203$ nats. We next examine two cases:*

(i) *If |X|=2, then it follows from (80) that $n^{*}=138$.*
(ii) *Consider a richer alphabet size of the i.i.d. samples where, e.g., |X|=100. By relying on the same universal lower bound $d^{*}$, which holds independently of the value of |X| (X can possibly be an infinite set), it follows from (80) that $n^{*}=4170$ is the minimal value such that the upper bound in (78) does not exceed $10^{-10}$.*

We close this discussion by providing numerical experimentation of the lower bound on the relative entropy in Theorem 2, and comparing this attainable lower bound (see Item b) of Theorem 2) with the following closed-form expressions for relative entropies:

(a) The relative entropy between real-valued Gaussian distributions is given by
  (82) $$\begin{array}{c} D\left(N\left(m_{P},\sigma_{P}^{2}\right)\;∥\;N\left(m_{Q},\sigma_{Q}^{2}\right)\right)=\log\frac{\sigma_{Q}}{\sigma_{P}}+\frac{1}{2}\left[\frac{{\left(m_{P}-m_{Q}\right)}^{2}+\sigma_{P}^{2}}{\sigma_{Q}^{2}}-1\right]\;\log e. \end{array}$$
(b) Let $E_{\mu }$ denote a random variable which is exponentially distributed with mean μ>0; its probability density function is given by
  (83) $$\begin{array}{c} e_{\mu }\left(x\right)=\frac{1}{\mu }\;e^{-x/\mu }\;1\left\{x\geq 0\right\}. \end{array}$$
  Then, for $a_{1},a_{2}>0$ and $d_{1},d_{2}\in R$,
  (84) $$D\left(E_{a_{1}}+d_{1}∥E_{a_{2}}+d_{2}\right)=\{\begin{array}{ll} \log\frac{a_{2}}{a_{1}}+\frac{d_{1}+a_{1}-d_{2}-a_{2}}{a_{2}}\;\log e, & \;d_{1}\geq d_{2},\\ \infty , & \;d_{1}<d_{2}. \end{array}$$
  In this case, the means under *P* and *Q* are $m_{P}=d_{1}+a_{1}$ and $m_{Q}=d_{2}+a_{2}$, respectively, and the variances are $\sigma_{P}^{2}=a_{1}^{2}$ and $\sigma_{Q}^{2}=a_{2}^{2}$. Hence, for obtaining the required means and variances, set
  (85) $$\begin{array}{c} a_{1}=\sigma_{P},\;a_{2}=\sigma_{Q},\;d_{1}=m_{P}-\sigma_{P},\;d_{2}=m_{Q}-\sigma_{Q}. \end{array}$$

**Example** **3.** *We compare numerically the attainable lower bound on the relative entropy, as it given in (40), with the two relative entropies in (82) and (84):*

(i) *If $\left(m_{P},m_{Q},\sigma_{P}^{2},\sigma_{Q}^{2}\right)=\left(45,40,20,20\right)$, then the lower bound in (40) is equal to 0.521 nats, and the two relative entropies in (82) and (84) are equal to 0.625 and 1.118 nats, respectively.*
(ii) *If $\left(m_{P},m_{Q},\sigma_{P}^{2},\sigma_{Q}^{2}\right)=\left(50,35,10,20\right)$, then the lower bound in (40) is equal to 2.332 nats, and the two relative entropies in (82) and (84) are equal to 5.722 and 3.701 nats, respectively.*

### 4.3. Strong Data–Processing Inequalities and Maximal Correlation

The information contraction is a fundamental concept in information theory. The contraction of *f*-divergences through channels is captured by data–processing inequalities, which can be further tightened by the derivation of SDPIs with channel-dependent or source-channel dependent contraction coefficients (see, e.g., [26,46,47,48,49,50,51,52]).

We next provide necessary definitions which are relevant for the presentation in this subsection.

**Definition** **7.** *Let $Q_{X}$ be a probability distribution which is defined on a set X, and that is not a point mass, and let $W_{Y|X}:X\to Y$ be a stochastic transformation. The contraction coefficient for f-divergences is defined as*

(86) $$\begin{array}{c} \mu_{f}\left(Q_{X},W_{Y|X}\right):=\underset{P_{X}:\;D_{f}\left(P_{X}∥Q_{X}\right)\in \left(0,\infty \right)}{\sup}\;\frac{D_{f}\left(P_{Y}∥Q_{Y}\right)}{D_{f}\left(P_{X}∥Q_{X}\right)}, \end{array}$$

*where, for all y∈Y,*

(87) $$\begin{array}{cc} \; & P_{Y}\left(y\right)=\left(P_{X}W_{Y|X}\right)\;\left(y\right):=\int_{X}dP_{X}\left(x\right)\;W_{Y|X}\left(y|x\right), \end{array}$$

(88) $$\begin{array}{cc} \; & Q_{Y}\left(y\right)=\left(Q_{X}W_{Y|X}\right)\;\left(y\right):=\int_{X}dQ_{X}\left(x\right)\;W_{Y|X}\left(y|x\right). \end{array}$$

*The notation in (87) and (88) is consistent with the standard notation used in information theory (see, e.g., the first displayed equation after (3.2) in [53]).*

The derivation of good upper bounds on contraction coefficients for *f*-divergences, which are strictly smaller than 1, lead to SDPIs. These inequalities find their applications, e.g., in studying the exponential convergence rate of an irreducible, time-homogeneous and reversible discrete-time Markov chain to its unique invariant distribution over its state space (see, e.g., [49] [Section 2.4.3] and [50] [Section 2]). It is in sharp contrast to DPIs which do not yield convergence to stationarity at any rate. We return to this point later in this subsection, and determine the exact convergence rate to stationarity under two parametric families of *f*-divergences.

We next rely on Theorem 1 to obtain upper bounds on the contraction coefficients for the following *f*-divergences.

**Definition** **8.** *For α∈(0,1], the α-skew K-divergence is given by*

(89) $$\begin{array}{c} K_{\alpha }\left(P∥Q\right):=D\left(P\;∥\;(1-\alpha )P+\alpha Q\right), \end{array}$$

*and, for α∈[0,1], let*

(90)Sα(P∥Q):=αDP∥(1−α)P+αQ+(1−α)DQ∥(1−α)P+αQ(91)=αKα(P∥Q)+(1−α)K1−α(Q∥P),

*with the convention that $K_{0}\left(P∥Q\right)\equiv 0$ (by a continuous extension at α=0 in (89)). These divergence measures are specialized to the relative entropies:*

(92) $$\begin{array}{cc} K_{1}\left(P∥Q\right)=D\left(P∥Q\right)=S_{1}\left(P∥Q\right), & \;S_{0}\left(P∥Q\right)=D\left(Q∥P\right), \end{array}$$

*and $S_{\frac{1}{2}}\left(P∥Q\right)$ is the Jensen–Shannon divergence [54,55,56] (also known as the capacitory discrimination [57]):*

(93)S12(P∥Q)=12DP∥12(P+Q)+12DQ∥12(P+Q)(94)=H12(P+Q)−12H(P)−12H(Q):=JS(P∥Q).

*It can be verified that the divergence measures in (89) and (90) are f-divergences:*

(95) $$\begin{array}{cc} \; & K_{\alpha }\left(P∥Q\right)=D_{k_{\alpha }}\left(P∥Q\right),\;\alpha \in \left(0,1\right], \end{array}$$

(96) $$\begin{array}{cc} \; & S_{\alpha }\left(P∥Q\right)=D_{s_{\alpha }}\left(P∥Q\right),\;\;\alpha \in \left[0,1\right], \end{array}$$

*with*

(97)kα(t):=tlogt−tlogα+(1−α)t,t>0,α∈(0,1],(98)sα(t):=αtlogt−αt+1−αlogα+(1−α)t(99)=αkα(t)+(1−α)tk1−α1t,t>0,α∈[0,1],

*where $k_{\alpha }\left(\cdot \right)$ and $s_{\alpha }\left(\cdot \right)$ are strictly convex functions on (0,∞), and vanish at 1.*

**Remark** **9.** *The α-skew K-divergence in (89) is considered in [55] and [58] [(13)] (including pointers in the latter paper to its utility). The divergence in (90) is akin to Lin’s measure in [55] [(4.1)], the asymmetric α-skew Jensen–Shannon divergence in [58] [(11)–(12)], the symmetric α-skew Jensen–Shannon divergence in [58] [(16)], and divergence measures in [59] which involve arithmetic and geometric means of two probability distributions. Properties and applications of quantum skew divergences are studied in [19] and references therein.*

**Theorem** **6.** *The f-divergences in (89) and (90) satisfy the following integral identities, which are expressed in terms of the Györfi–Vajda divergence in (17):*

(100) $$\begin{array}{cc} \; & \frac{1}{\log e}\;K_{\alpha }\left(P∥Q\right)=\int_{0}^{\alpha }sD_{\phi_{s}}\left(P∥Q\right)\;ds,\;\alpha \in \left(0,1\right], \end{array}$$

(101) $$\begin{array}{cc} \; & \frac{1}{\log e}\;S_{\alpha }\left(P∥Q\right)=\int_{0}^{1}g_{\alpha }\left(s\right)\;D_{\phi_{s}}\left(P∥Q\right)\;ds,\;\alpha \in \left[0,1\right], \end{array}$$

*with*

(102) $$\begin{array}{c} g_{\alpha }\left(s\right):=\alpha s\;1\left\{s\in (0,\alpha ]\right\}+\left(1-\alpha \right)\left(1-s\right)\;1\left\{s\in [\alpha ,1)\right\},\;\left(\alpha ,s\right)\in \left[0,1\right]\times \left[0,1\right]. \end{array}$$

*Moreover, the contraction coefficients for these f-divergences are related as follows:*

(103) $$\begin{array}{cc} \mu_{\chi^{2}}\left(Q_{X},W_{Y|X}\right) & \leq \mu_{k_{\alpha }}\left(Q_{X},W_{Y|X}\right)\leq \underset{s\in (0,\alpha ]}{\sup}\mu_{\phi_{s}}\left(Q_{X},W_{Y|X}\right),\;\alpha \in \left(0,1\right], \end{array}$$

(104) $$\begin{array}{cc} \mu_{\chi^{2}}\left(Q_{X},W_{Y|X}\right) & \leq \mu_{s_{\alpha }}\left(Q_{X},W_{Y|X}\right)\leq \underset{s\in (0,1)}{\sup}\mu_{\phi_{s}}\left(Q_{X},W_{Y|X}\right),\;\;\alpha \in \left[0,1\right], \end{array}$$

*where $\mu_{\chi^{2}}\left(Q_{X},W_{Y|X}\right)$ denotes the contraction coefficient for the chi-squared divergence.*

**Proof.**  See Section 5.8. □

**Remark** **10.** *The upper bounds on the contraction coefficients for the parametric f-divergences in (89) and (90) generalize the upper bound on the contraction coefficient for the relative entropy in [51] [Theorem III.6] (recall that $K_{1}\left(P∥Q\right)=D\left(P∥Q\right)=S_{1}\left(P∥Q\right)$), so the upper bounds in Theorem 6 are specialized to the latter bound at α=1.*

**Corollary** **7.** *Let*

(105) $$\begin{array}{c} \mu_{\chi^{2}}\left(W_{Y|X}\right):=\underset{Q}{\sup}\mu_{\chi^{2}}\left(Q_{X},W_{Y|X}\right), \end{array}$$

*where the supremum on the right side is over all probability measures $Q_{X}$ defined on X. Then,*

(106) $$\begin{array}{cc} \mu_{\chi^{2}}\left(Q_{X},W_{Y|X}\right) & \leq \mu_{k_{\alpha }}\left(Q_{X},W_{Y|X}\right)\leq \mu_{\chi^{2}}\left(W_{Y|X}\right),\;\alpha \in \left(0,1\right], \end{array}$$

(107) $$\begin{array}{cc} \mu_{\chi^{2}}\left(Q_{X},W_{Y|X}\right) & \leq \mu_{s_{\alpha }}\left(Q_{X},W_{Y|X}\right)\leq \mu_{\chi^{2}}\left(W_{Y|X}\right),\;\;\alpha \in \left[0,1\right]. \end{array}$$

**Proof.**  See Section 5.9. □

**Example** **4.** *Let $Q_{X}=\mathrm{Bernoulli}\left(\frac{1}{2}\right)$, and let $W_{Y|X}$ correspond to a binary symmetric channel (BSC) with crossover probability ε. Then, $\mu_{\chi^{2}}\left(Q_{X},W_{Y|X}\right)=\mu_{\chi^{2}}\left(W_{Y|X}\right)={\left(1-2\varepsilon \right)}^{2}$. The upper and lower bounds on $\mu_{k_{\alpha }}\left(Q_{X},W_{Y|X}\right)$ and $\mu_{s_{\alpha }}\left(Q_{X},W_{Y|X}\right)$ in (106) and (107) match for all α, and they are all equal to ${\left(1-2\varepsilon \right)}^{2}$.*

The upper bound on the contraction coefficients in Corollary 7 is given by $\mu_{\chi^{2}}\left(W_{Y|X}\right)$, whereas the lower bound is given by $\mu_{\chi^{2}}\left(Q_{X},W_{Y|X}\right)$, which depends on the input distribution $Q_{X}$. We next provide alternative upper bounds on the contraction coefficients for the considered (parametric) *f*-divergences, which, similarly to the lower bound, scale like $\mu_{\chi^{2}}\left(Q_{X},W_{Y|X}\right)$. Although the upper bound in Corollary 7 may be tighter in some cases than the alternative upper bounds which are next presented in Proposition 3 (and in fact, the former upper bound may be even achieved with equality as in Example 4), the bounds in Proposition 3 are used shortly to determine the exponential rate of the convergence to stationarity of a type of Markov chains.

**Proposition** **3.** *For all α∈(0,1],*

(108)μχ2(QX,WY|X)≤μkα(QX,WY|X)≤1αQmin·μχ2(QX,WY|X),(109)μχ2(QX,WY|X)≤μsα(QX,WY|X)≤(1−α)loge1α+2α−1(1−3α+3α2)Qmin·μχ2(QX,WY|X),

*where $Q_{\min}$ denotes the minimal positive mass of the input distribution $Q_{X}$.*

**Proof.**  See Section 5.10. □

**Remark** **11.** *In view of (92), at α=1, (108) and (109) specialize to an upper bound on the contraction coefficient of the relative entropy (KL divergence) as a function of the contraction coefficient of the chi-squared divergence. In this special case, both (108) and (109) give*

(110) $$\begin{array}{c} \mu_{\chi^{2}}\left(Q_{X},W_{Y|X}\right)\leq \mu_{\mathrm{KL}}\left(Q_{X},W_{Y|X}\right)\leq \frac{1}{Q_{\min}}\cdot \mu_{\chi^{2}}\left(Q_{X},W_{Y|X}\right), \end{array}$$

*which then coincides with [48] [Theorem 10].*

We next apply Proposition 3 to consider the convergence rate to stationarity of Markov chains by the introduced *f*-divergences in Definition 8. The next result follows [49] [Section 2.4.3], and it provides a generalization of the result there.

**Theorem** **7.** *Consider a time-homogeneous, irreducible, and reversible discrete-time Markov chain with a finite state space X, let W be its probability transition matrix, and $Q_{X}$ be its unique stationary distribution (reversibility means that $Q_{X}\left(x\right){\left[W\right]}_{x,y}=Q_{X}\left(y\right){\left[W\right]}_{y,x}$ for all x,y∈X). Let $P_{X}$ be an initial probability distribution over X. Then, for all α∈(0,1] and n∈N,*

(111)Kα(PXWn∥QX)≤μkα(QX,Wn)Kα(PX∥QX),(112)Sα(PXWn∥QX)≤μsα(QX,Wn)Sα(PX∥QX),

*and the contraction coefficients on the right sides of (111) and (112) scale like the n-th power of the contraction coefficient for the chi-squared divergence as follows:*

(113)μχ2(QX,W)n≤μkα(QX,Wn)≤1αQmin·μχ2(QX,W)n,(114)μχ2(QX,W)n≤μsα(QX,Wn)≤(1−α)loge1α+2α−1(1−3α+3α2)Qmin·μχ2(QX,W)n.

**Proof.**  Inequalities (111) and (112) hold since $Q_{X}W^{n}=Q_{X}$, for all n∈N, and due to Definition 7 and (95) and (96). Inequalities (113) and (114) hold by Proposition 3, and due to the reversibility of the Markov chain which implies that (see [49] [Equation (2.92)])

(115) $$\begin{array}{c} \mu_{\chi^{2}}\left(Q_{X},W^{n}\right)={\left(\mu_{\chi^{2}}\left(Q_{X},W\right)\right)}^{n},\;n\in N. \end{array}$$

 □

In view of (113) and (114), Theorem 7 readily gives the following result on the exponential decay rate of the upper bounds on the divergences on the left sides of (111) and (112).

**Corollary** **8.** *For all α∈(0,1],*

(116) $$\begin{array}{c} \lim_{n\to \infty }{\left(\mu_{k_{\alpha }}\left(Q_{X},W^{n}\right)\right)}^{1/n}=\mu_{\chi^{2}}\left(Q_{X},W\right)=\lim_{n\to \infty }{\left(\mu_{s_{\alpha }}\left(Q_{X},W^{n}\right)\right)}^{1/n}. \end{array}$$

**Remark** **12.** *Theorem 7 and Corollary 8 generalize the results in [49] [Section 2.4.3], which follow as a special case at α=1 (see (92)).*

We end this subsection by considering maximal correlations, which are closely related to the contraction coefficient for the chi-squared divergence.

**Definition** **9.** *The maximal correlation between two random variables X and Y is defined as*

(117) $$\begin{array}{c} \rho_{m}\left(X;Y\right):=\underset{f,g}{\sup}\;E\left[f\left(X\right)g\left(Y\right)\right], \end{array}$$

*where the supremum is taken over all real-valued functions f and g such that*

(118) $$\begin{array}{c} E\left[f\left(X\right)\right]=E\left[g\left(Y\right)\right]=0,\;E\left[f^{2}\left(X\right)\right]\leq 1,\;E\left[g^{2}\left(Y\right)\right]\leq 1. \end{array}$$

It is well-known [60] that, if $X∼Q_{X}$ and $Y∼Q_{Y}=Q_{X}W_{Y|X}$, then the contraction coefficient for the chi-squared divergence $\mu_{\chi^{2}}\left(Q_{X},W_{Y|X}\right)$ is equal to the square of the maximal correlation between the random variables *X* and *Y*, i.e.,

(119) $$\begin{array}{c} \rho_{m}\left(X;Y\right)=\sqrt{\mu_{\chi^{2}}\left(Q_{X},W_{Y|X}\right)}. \end{array}$$

A simple application of Corollary 1 and (119) gives the following result.

**Proposition** **4.** *In the setting of Definition 7, for s∈[0,1], let $X_{s}∼\left(1-s\right)P_{X}+sQ_{X}$ and $Y_{s}∼\left(1-s\right)P_{Y}+sQ_{Y}$ with $P_{X}\neq Q_{X}$ and $P_{X}\ll \gg Q_{X}$. Then, the following inequality holds:*

(120) $$\begin{array}{c} \underset{s\in [0,1]}{\sup}\rho_{m}\left(X_{s};Y_{s}\right)\geq \max\left\{\sqrt{\frac{D(P_{Y}∥Q_{Y})}{D(P_{X}∥Q_{X})}},\;\sqrt{\frac{D(Q_{Y}∥P_{Y})}{D(Q_{X}∥P_{X})}}\right\}. \end{array}$$

**Proof.**  See Section 5.11. □

## 5. Proofs

This section provides proofs of the results in Section 3 and Section 4.

### 5.1. Proof of Theorem 1

*Proof of (22)*: We rely on an integral representation of the logarithm function (on base e):

(121) $$\begin{array}{cc} \log_{e}x\; & =\int_{0}^{1}\frac{x-1}{x+(1-x)v}\;dv,\;\forall \;x>0. \end{array}$$

Let μ be a dominating measure of *P* and *Q* (i.e., P,Q≪μ), and let $p:=\frac{dP}{d\mu }$, $q:=\frac{dQ}{d\mu }$, and

(122) $$\begin{array}{c} r_{\lambda }:=\frac{dR_{\lambda }}{d\mu }=\left(1-\lambda \right)p+\lambda q,\;\forall \;\lambda \in \left[0,1\right], \end{array}$$

where the last equality is due to (21). For all λ∈[0,1],

(123)1logeD(P∥Rλ)=∫plogeprλdμ(124)=∫01∫p(p−rλ)p+v(rλ−p)dμdv,

where (124) holds due to (121) with $x:=\frac{p}{r_{\lambda }}$, and by swapping the order of integration. The inner integral on the right side of (124) satisfies, for all v∈(0,1],

(125)∫p(p−rλ)p+v(rλ−p)dμ=∫(p−rλ)1+v(p−rλ)p+v(rλ−p)dμ(126)=∫(p−rλ)dμ+v∫(p−rλ)2p+v(rλ−p)dμ(127)=v∫(p−rλ)2(1−v)p+vrλdμ(128)=1v∫p−(1−v)p+vrλ2(1−v)p+vrλdμ(129)=1vχ2P∥(1−v)P+vRλ,

where (127) holds since ∫pdμ=1, and $\int r_{\lambda }\;d\mu =1$. From (21), for all (λ,v)∈[0,1]×[0,1],

(130) $$\begin{array}{c} \left(1-v\right)P+vR_{\lambda }=\left(1-\lambda v\right)P+\lambda v\;Q=R_{\lambda v}. \end{array}$$

The substitution of (130) into the right side of (129) gives that, for all (λ,v)∈[0,1]×(0,1],

(131) $$\begin{array}{c} \int \frac{p(p-r_{\lambda })}{p+v(r_{\lambda }-p)}\;d\mu =\frac{1}{v}\;\chi^{2}\left(P∥R_{\lambda v}\right). \end{array}$$

Finally, substituting (131) into the right side of (124) gives that, for all λ∈(0,1],

(132)1logeD(P∥Rλ)=∫011vχ2(P∥Rλv)dv(133)=∫0λ1sχ2(P∥Rs)ds,

where (133) holds by the transformation s:=λv. Equality (133) also holds for λ=0 since we have $D\left(P∥R_{0}\right)=D\left(P∥P\right)=0$.

*Proof of (23)*: For all s∈(0,1],

χ2(P∥Q)=∫(p−q)2qdμ(134)=1s2∫sp+(1−s)q−q2qdμ(135)=1s2∫r1−s−q2qdμ(136)=1s2χ2R1−s∥Q,

where (135) holds due to (122). From (136), it follows that for all λ∈[0,1],

(137) $$\begin{array}{c} \int_{0}^{\lambda }\frac{1}{s}\;\chi^{2}\left(R_{1-s}\;∥\;Q\right)\;ds=\int_{0}^{\lambda }s\;ds\;\;\chi^{2}\left(P∥Q\right)=\frac{1}{2}\;\lambda^{2}\;\chi^{2}\left(P∥Q\right). \end{array}$$

### 5.2. Proof of Proposition 1

(a) *Simple Proof of Pinsker’s Inequality*: By [61] or [62] [(58)],
  (138) $$\chi^{2}\left(P∥Q\right)\geq \{\begin{array}{ll} {\left|P-Q\right|}^{2}, & \mathrm{if}\;|P-Q|\in [0,1],\\ \frac{\left|P-Q\right|}{2-|P-Q|}, & \mathrm{if}\;|P-Q|\in (1,2]. \end{array}$$
  We need the weaker inequality $\chi^{2}\left(P∥Q\right)\geq {\left|P-Q\right|}^{2}$, proved by the Cauchy–Schwarz inequality:
  (139)χ2(P∥Q)=∫(p−q)2qdμ∫qdμ(140)≥∫|p−q|q·qdμ2(141)=|P−Q|2.
  By combining (24) and (139)–(141), it follows that
  (142)1logeD(P∥Q)=∫01χ2(P∥(1−s)P+sQ)dss(143)≥∫01|P−(1−s)P+sQ|2dss(144)=∫01s|P−Q|2ds(145)=12|P−Q|2.
(b) *Proof of (30) and its local tightness*:
  (146)1logeD(P∥Q)=∫01χ2(P∥(1−s)P+sQ)dss(147)=∫01∫p−((1−s)p+sq)2(1−s)p+sqdμdss(148)=∫01∫s(p−q)2(1−s)p+sqdμds(149)≤∫01∫s(p−q)21−sp+sqdμds(150)=∫01s2ds∫(p−q)2qdμ+∫01s(1−s)ds∫(p−q)2pdμ(151)=13χ2(P∥Q)+16χ2(Q∥P),
  where (146) is (24), and (149) holds due to Jensen’s inequality and the convexity of the hyperbola.
  We next show the local tightness of inequality (30) by proving that (31) yields (32). Let $\left\{P_{n}\right\}$ be a sequence of probability measures, defined on a measurable space (X,F), and assume that $\left\{P_{n}\right\}$ converges to a probability measure *P* in the sense that (31) holds. In view of [16] [Theorem 7] (see also [15] [Section 4.F] and [63]), it follows that
  (152) $$\begin{array}{c} \lim_{n\to \infty }D\left(P_{n}∥P\right)=\lim_{n\to \infty }\chi^{2}\left(P_{n}∥P\right)=0, \end{array}$$
  and
  (153)limn→∞D(Pn∥P)χ2(Pn∥P)=12loge,(154)limn→∞χ2(Pn∥P)χ2(P∥Pn)=1,
  which therefore yields (32).
(c) *Proof of (33) and (34)*: The proof of (33) relies on (28) and the following lemma.
  **Lemma 1.**  *For all s,θ∈(0,1),*
  (155) $$\begin{array}{c} \frac{D_{\phi_{s}}\left(P∥Q\right)}{D_{\phi_{\theta }}\left(P∥Q\right)}\geq \min\left\{\frac{1-\theta }{1-s},\frac{\theta }{s}\right\}. \end{array}$$
  **Proof.**  (156)Dϕs(P∥Q)=∫(p−q)2(1−s)p+sqdμ(157)=∫(p−q)2(1−θ)p+θq(1−θ)p+θq(1−s)p+sqdμ(158)≥min1−θ1−s,θs∫(p−q)2(1−θ)p+θqdμ(159)=min1−θ1−s,θsDϕθ(P∥Q).
   □
  From (28) and (155), for all θ∈(0,1),
  (160)1logeD(P∥Q)=∫0θsDϕs(P∥Q)ds+∫θ1sDϕs(P∥Q)ds(161)≥∫0θs(1−θ)1−s·Dϕθ(P∥Q)ds+∫θ1θDϕθ(P∥Q)ds(162)=−θ+loge11−θ(1−θ)Dϕθ(P∥Q)+θ(1−θ)Dϕθ(P∥Q)(163)=(1−θ)loge11−θDϕθ(P∥Q).
  This proves (33). Furthermore, under the assumption in (31), for all θ∈[0,1],
  (164)limn→∞D(P∥Pn)Dϕθ(P∥Pn)=limn→∞D(P∥Pn)χ2(P∥Pn)limn→∞χ2(P∥Pn)Dϕθ(P∥Pn)(165)=12loge·2ϕθ″(1)(166)=12loge,
  where (165) holds due to (153) and the local behavior of *f*-divergences [63], and (166) holds due to (17) which implies that $\phi_{\theta }^{\prime \prime }\left(1\right)=2$ for all θ∈[0,1]. This proves (34).
(d) *Proof of (35)*: From (24), we get
  (167)1logeD(P∥Q)=∫01χ2(P∥(1−s)P+sQ)dss(168)=∫01χ2(P∥(1−s)P+sQ)−s2χ2(P∥Q)dss+∫01sdsχ2(P∥Q)(169)=∫01χ2(P∥(1−s)P+sQ)−s2χ2(P∥Q)dss+12χ2(P∥Q).
  Referring to the integrand of the first term on the right side of (169), for all s∈(0,1],
  1sχ2(P∥(1−s)P+sQ)−s2χ2(P∥Q)(170)=s∫(p−q)21(1−s)p+sq−1qdμ(171)=s(1−s)∫(q−p)3q(1−s)p+sqdμ(172)=s(1−s)∫|q−p|·|q−p|q·q−pp+s(q−p)︸≤1s1{q≥p}dμ(173)≤(1−s)∫(q−p)1{q≥p}dμ(174)=12(1−s)|P−Q|,
  where the last equality holds since the equality ∫(q−p)dμ=0 implies that
  (175)∫(q−p)1{q≥p}dμ=∫(p−q)1{p≥q}dμ(176)=12∫|p−q|dμ=12|P−Q|.
  From (170)–(174), an upper bound on the right side of (169) results. This gives
  (177)1logeD(P∥Q)≤12∫01(1−s)ds|P−Q|+12χ2(P∥Q)(178)=14|P−Q|+12χ2(P∥Q).
  It should be noted that [15] [Theorem 2(a)] shows that inequality (35) is tight. To that end, let ε∈(0,1), and define probability measures $P_{\varepsilon }$ and $Q_{\varepsilon }$ on the set A={0,1} with $P_{\varepsilon }\left(1\right)=\varepsilon^{2}$ and $Q_{\varepsilon }\left(1\right)=\varepsilon$. Then,
  (179) $$\begin{array}{cc} \; & \lim_{\varepsilon ↓0}\frac{\frac{1}{\log e}\;D\left(P_{\varepsilon }∥Q_{\varepsilon }\right)}{\frac{1}{4}\;\left|P_{\varepsilon }-Q_{\varepsilon }\right|+\frac{1}{2}\;\chi^{2}\left(P_{\varepsilon }∥Q_{\varepsilon }\right)}=1. \end{array}$$

### 5.3. Proof of Theorem 2

We first prove Item (a) in Theorem 2. In view of the Hammersley–Chapman–Robbins lower bound on the $\chi^{2}$ divergence, for all λ∈[0,1]

(180) $$\begin{array}{c} \chi^{2}\left(P∥(1-\lambda )P+\lambda Q\right)\geq \frac{{\left(E\left[X\right]-E\left[Z_{\lambda }\right]\right)}^{2}}{\mathrm{Var}(Z_{\lambda })}, \end{array}$$

where X∼P, Y∼Q and $Z_{\lambda }∼R_{\lambda }:=\left(1-\lambda \right)P+\lambda Q$ is defined by

(181) $$Z_{\lambda }:=\{\begin{array}{ll} X, & \;\mathrm{with}\;\mathrm{probability}\;1-\lambda ,\\ Y, & \;\mathrm{with}\;\mathrm{probability}\;\lambda . \end{array}$$

For λ∈[0,1],

(182) $$\begin{array}{c} E\left[Z_{\lambda }\right]=\left(1-\lambda \right)m_{P}+\lambda m_{Q}, \end{array}$$

and it can be verified that

(183) $$\begin{array}{c} \mathrm{Var}\left(Z_{\lambda }\right)=\left(1-\lambda \right)\sigma_{P}^{2}+\lambda \sigma_{Q}^{2}+\lambda \left(1-\lambda \right){\left(m_{P}-m_{Q}\right)}^{2}. \end{array}$$

We now rely on identity (24)

(184) $$\begin{array}{cc} \frac{1}{\log e}\;D\left(P∥Q\right) & =\int_{0}^{1}\chi^{2}\left(P∥\left(1-\lambda \right)P+\lambda Q\right)\;\frac{d\lambda }{\lambda } \end{array}$$

to get a lower bound on the relative entropy. Combining (180), (183) and (184) yields

(185) $$\begin{array}{c} \frac{1}{\log e}\;D\left(P∥Q\right)\geq {\left(m_{P}-m_{Q}\right)}^{2}\int_{0}^{1}\frac{\lambda }{\left(1-\lambda \right)\sigma_{P}^{2}+\lambda \sigma_{Q}^{2}+\lambda \left(1-\lambda \right){\left(m_{P}-m_{Q}\right)}^{2}}\;d\lambda . \end{array}$$

From (43) and (44), we get

(186) $$\begin{array}{c} \int_{0}^{1}\frac{\lambda }{\left(1-\lambda \right)\sigma_{P}^{2}+\lambda \sigma_{Q}^{2}+\lambda \left(1-\lambda \right){\left(m_{P}-m_{Q}\right)}^{2}}\;d\lambda =\int_{0}^{1}\frac{\lambda }{\left(\alpha -a\lambda )(\beta +a\lambda \right)}\;d\lambda , \end{array}$$

where

(187) $$\begin{array}{cc} \alpha & :=\sqrt{\sigma_{P}^{2}+\frac{b^{2}}{4a^{2}}}+\frac{b}{2a}, \end{array}$$

(188) $$\begin{array}{cc} \beta & :=\sqrt{\sigma_{P}^{2}+\frac{b^{2}}{4a^{2}}}-\frac{b}{2a}. \end{array}$$

By using the partial fraction decomposition of the integrand on the right side of (186), we get (after multiplying both sides of (185) by loge)

(189)D(P∥Q)≥(mP−mQ)2a2αα+βlogαα−a+βα+βlogββ+a(190)=αα+βlogαα−a+βα+βlogββ+a(191)=d(αα+βα−aα+β,

where (189) holds by integration since α−aλ and β+aλ are both non-negative for all λ∈[0,1]. To verify the latter claim, it should be noted that (43) and the assumption that $m_{P}\neq m_{Q}$ imply that a≠0. Since α,β>0, it follows that, for all λ∈[0,1], either α−aλ>0 or β+aλ>0 (if a<0, then the former is positive, and, if a>0, then the latter is positive). By comparing the denominators of both integrands on the left and right sides of (186), it follows that (α−aλ)(β+aλ)≥0 for all λ∈[0,1]. Since the product of α−aλ and β+aλ is non-negative and at least one of these terms is positive, it follows that α−aλ and β+aλ are both non-negative for all λ∈[0,1]. Finally, (190) follows from (43).

If $m_{P}-m_{Q}\to 0$ and $\sigma_{P}\neq \sigma_{Q}$, then it follows from (43) and (44) that a→0 and $b\to \sigma_{P}^{2}-\sigma_{Q}^{2}\neq 0$. Hence, from (187) and (188), $\alpha \geq \left|\frac{b}{a}\right|\to \infty$ and β→0, which implies that the lower bound on D(P∥Q) in (191) tends to zero.

Letting $r:=\frac{\alpha }{\alpha +\beta }$ and $s:=\frac{\alpha -a}{\alpha +\beta }$, we obtain that the lower bound on D(P∥Q) in (40) holds. This bound is consistent with the expressions of *r* and *s* in (41) and (42) since, from (45), (187) and (188),

(192) $$\begin{array}{cc} r & =\frac{\alpha }{\alpha +\beta }=\frac{v+\frac{b}{2a}}{2v}=\frac{1}{2}+\frac{b}{4av}, \end{array}$$

(193) $$\begin{array}{cc} s & =\frac{\alpha -a}{\alpha +\beta }=r-\frac{a}{\alpha +\beta }=r-\frac{a}{2v}. \end{array}$$

It should be noted that r,s∈[0,1]. First, from (187) and (188), α and β are positive if $\sigma_{P}\neq 0$, which yields $r=\frac{\alpha }{\alpha +\beta }\in \left(0,1\right)$. We next show that s∈[0,1]. Recall that α−aλ and β+aλ are both non-negative for all λ∈[0,1]. Setting λ=1 yields α≥a, which (from (193)) implies that s≥0. Furthermore, from (193) and the positivity of α+β, it follows that s≤1 if and only if β≥−a. The latter holds since β+aλ≥0 for all λ∈[0,1] (in particular, for λ=1). If $\sigma_{P}=0$, then it follows from (41)–(45) that $v=\frac{b}{2|a|}$, $b=a^{2}+\sigma_{Q}^{2}$, and (recall that a≠0)

(i) If a>0, then $v=\frac{b}{2a}$ implies that $r=\frac{1}{2}+\frac{b}{4av}=1$, and $s=r-\frac{a}{2v}=1-\frac{a^{2}}{b}=\frac{\sigma_{Q}^{2}}{\sigma_{Q}^{2}+a^{2}}\in \left[0,1\right]$;
(ii) If a<0, then $v=-\frac{b}{2a}$ implies that r=0, and $s=r-\frac{a}{2v}=\frac{a^{2}}{b}=\frac{a^{2}}{a^{2}+\sigma_{Q}^{2}}\in \left[0,1\right]$.

We next prove Item (b) in Theorem 2 (i.e., the achievability of the lower bound in (40)). To that end, we provide a technical lemma, which can be verified by the reader.

**Lemma** **2.** *Let r,s be given in (41)–(45), and let $u_{1,2}$ be given in (47). Then,*

(194) $$\begin{array}{cc} \; & \left(s-r\right)\left(u_{1}-u_{2}\right)=m_{Q}-m_{P}, \end{array}$$

(195) $$\begin{array}{cc} \; & u_{1}+u_{2}=m_{P}+m_{Q}+\frac{\sigma_{Q}^{2}-\sigma_{P}^{2}}{m_{Q}-m_{P}}. \end{array}$$

Let X∼P and Y∼Q be defined on a set $U=\{u_{1},u_{2}\}$ (for the moment, the values of $u_{1}$ and $u_{2}$ are not yet specified) with $P[X=u_{1}]=r$, $P[X=u_{2}]=1-r$, $Q[Y=u_{1}]=s$, and $Q[Y=u_{2}]=1-s$. We now calculate $u_{1}$ and $u_{2}$ such that $E\left[X\right]=m_{P}$ and $\mathrm{Var}\left(X\right)=\sigma_{P}^{2}$. This is equivalent to

(196)ru1+(1−r)u2=mP,(197)ru12+(1−r)u22=mP2+σP2.

Substituting (196) into the right side of (197) gives

(198) $$\begin{array}{c} ru_{1}^{2}+\left(1-r\right)u_{2}^{2}={\left[ru_{1}+\left(1-r\right)u_{2}\right]}^{2}+\sigma_{P}^{2}, \end{array}$$

which, by rearranging terms, also gives

(199) $$\begin{array}{c} u_{1}-u_{2}=\pm \sqrt{\frac{\sigma_{P}^{2}}{r(1-r)}}. \end{array}$$

Solving simultaneously (196) and (199) gives

(200) $$\begin{array}{cc} \; & u_{1}=m_{P}\pm \sqrt{\frac{\left(1-r\right)\sigma_{P}^{2}}{r}}, \end{array}$$

(201) $$\begin{array}{cc} \; & u_{2}=m_{P}\mp \sqrt{\frac{r\sigma_{P}^{2}}{1-r}}. \end{array}$$

We next verify that, by setting $u_{1,2}$ as in (47), one also gets (as desired) that $E\left[Y\right]=m_{Q}$ and $\mathrm{Var}\left(Y\right)=\sigma_{Q}^{2}$. From Lemma 2, and, from (196) and (197), we have

(202)E[Y]=su1+(1−s)u2(203)=ru1+(1−r)u2+(s−r)(u1−u2)(204)=mP+(s−r)(u1−u2)=mQ,(205)E[Y2]=su12+(1−s)u22(206)=ru12+(1−r)u22+(s−r)(u12−u22)(207)=E[X2]+(s−r)(u1−u2)(u1+u2)(208)=mP2+σP2+(mQ−mP)mP+mQ+σQ2−σP2mQ−mP(209)=mQ2+σQ2.

By combining (204) and (209), we obtain $\mathrm{Var}\left(Y\right)=\sigma_{Q}^{2}$. Hence, the probability mass functions *P* and *Q* defined on $U=\{u_{1},u_{2}\}$ (with $u_{1}$ and $u_{2}$ in (47)) such that

(210) $$\begin{array}{c} P\left(u_{1}\right)=1-P\left(u_{2}\right)=r,\;Q\left(u_{1}\right)=1-Q\left(u_{2}\right)=s \end{array}$$

satisfy the equality constraints in (39), while also achieving the lower bound on D(P∥Q) that is equal to d(r∥s). It can be also verified that the second option where

(211) $$\begin{array}{c} u_{1}=m_{P}-\sqrt{\frac{\left(1-r\right)\sigma_{P}^{2}}{r}},\;u_{2}=m_{P}+\sqrt{\frac{r\sigma_{P}^{2}}{1-r}} \end{array}$$

does *not* yield the satisfiability of the conditions $E\left[Y\right]=m_{Q}$ and $\mathrm{Var}\left(Y\right)=\sigma_{Q}^{2}$, so there is only a unique pair of probability measures *P* and *Q*, defined on a two-element set that achieves the lower bound in (40) under the equality constraints in (39).

We finally prove Item (c) in Theorem 2. Let $m\in R,\sigma_{P}^{2}$, and $\sigma_{Q}^{2}$ be selected arbitrarily such that $\sigma_{Q}^{2}\geq \sigma_{P}^{2}$. We construct probability measures $P_{\varepsilon }$ and $Q_{\varepsilon }$, depending on a free parameter ε, with means $m_{P}=m_{Q}:=m$ and variances $\sigma_{P}^{2}$ and $\sigma_{Q}^{2}$, respectively (means and variances are independent of ε), and which are defined on a three-element set $U:=\{u_{1},u_{2},u_{3}\}$ as follows:

(212) $$\begin{array}{cc} \; & P_{\varepsilon }\left(u_{1}\right)=r,\;\;P_{\varepsilon }\left(u_{2}\right)=1-r,\;\;\;P_{\varepsilon }\left(u_{3}\right)=0, \end{array}$$

(213) $$\begin{array}{cc} \; & Q_{\varepsilon }\left(u_{1}\right)=s,\;Q_{\varepsilon }\left(u_{2}\right)=1-s-\varepsilon ,\;Q_{\varepsilon }\left(u_{3}\right)=\varepsilon , \end{array}$$

with ε>0. We aim to set the parameters $r,s,u_{1},u_{2}$ and $u_{3}$ (as a function of $m,\sigma_{P},\sigma_{Q}$ and ε) such that

(214) $$\begin{array}{c} \lim_{\varepsilon \to 0^{+}}\;D\left(P_{\varepsilon }∥Q_{\varepsilon }\right)=0. \end{array}$$

Proving (214) yields (48), while it also follows that the infimum on the left side of (48) can be restricted to probability measures which are defined on a three-element set.

In view of the constraints on the means and variances in (39), with equal means *m*, we get the following set of equations from (212) and (213):

(215) $$\begin{array}{c} \left\{\begin{array}{cc} \; & ru_{1}+\left(1-r\right)u_{2}=m,\\ \; & su_{1}+\left(1-s-\varepsilon \right)u_{2}+\varepsilon u_{3}=m,\\ \; & ru_{1}^{2}+\left(1-r\right)u_{2}^{2}=m^{2}+\sigma_{P}^{2},\\ \; & su_{1}^{2}+\left(1-s-\varepsilon \right)u_{2}^{2}+\varepsilon u_{3}^{2}=m^{2}+\sigma_{Q}^{2}. \end{array}\right. \end{array}$$

The first and second equations in (215) refer to the equal means under *P* and *Q*, and the third and fourth equations in (215) refer to the second moments in (39). Furthermore, in view of (212) and (213), the relative entropy is given by

(216) $$\begin{array}{c} D\left(P_{\varepsilon }∥Q_{\varepsilon }\right)=r\log\frac{r}{s}+\left(1-r\right)\log\frac{1-r}{1-s-\varepsilon }. \end{array}$$

Subtracting the square of the first equation in (215) from its third equation gives the equivalent set of equations

(217) $$\begin{array}{c} \left\{\begin{array}{cc} \; & ru_{1}+\left(1-r\right)u_{2}=m,\\ \; & su_{1}+\left(1-s-\varepsilon \right)u_{2}+\varepsilon u_{3}=m,\\ \; & r\left(1-r\right){\left(u_{1}-u_{2}\right)}^{2}=\sigma_{P}^{2},\\ \; & su_{1}^{2}+\left(1-s-\varepsilon \right)u_{2}^{2}+\varepsilon u_{3}^{2}=m^{2}+\sigma_{Q}^{2}. \end{array}\right. \end{array}$$

We next select $u_{1}$ and $u_{2}$ such that $u_{1}-u_{2}:=2\sigma_{P}$. Then, the third equation in (217) gives $r\left(1-r\right)=\frac{1}{4}$, so $r=\frac{1}{2}$. Furthermore, the first equation in (217) gives

(218) $$\begin{array}{cc} \; & u_{1}=m+\sigma_{P}, \end{array}$$

(219) $$\begin{array}{cc} \; & u_{2}=m-\sigma_{P}. \end{array}$$

Since *r*, $u_{1}$, and $u_{2}$ are independent of ε, so is the probability measure $P_{\varepsilon }:=P$. Combining the second equation in (217) with (218) and (219) gives

(220) $$\begin{array}{c} u_{3}=m-\left(1+\frac{2s-1}{\varepsilon }\right)\sigma_{P}. \end{array}$$

Substituting (218)–(220) into the fourth equation of (217) gives a quadratic equation for *s*, whose selected solution (such that *s* and $r=\frac{1}{2}$ be close for small ϵ>0) is equal to

(221) $$\begin{array}{c} s=\frac{1}{2}\left[1-\varepsilon +\sqrt{\left(\frac{\sigma_{Q}^{2}}{\sigma_{P}^{2}}-1+\varepsilon \right)\varepsilon }\;\right]. \end{array}$$

Hence, $s=\frac{1}{2}+O\left(\sqrt{\varepsilon }\right)$, which implies that s∈(0,1−ε) for sufficiently small ε>0 (as it is required in (213)). In view of (216), it also follows that $D(P∥Q_{\varepsilon })$ vanishes as we let ε tend to zero.

We finally outline an alternative proof, which refers to the case of equal means with arbitrarily selected $\sigma_{P}^{2}$ and $\sigma_{Q}^{2}$. Let $\left(\sigma_{P}^{2},\sigma_{Q}^{2}\right)\in {\left(0,\infty \right)}^{2}$. We next construct a sequence of pairs of probability measures $\left\{\left(P_{n},Q_{n}\right)\right\}$ with zero mean and respective variances $\left(\sigma_{P}^{2},\sigma_{Q}^{2}\right)$ for which $D(P_{n}∥Q_{n})\to 0$ as n→∞ (without any loss of generality, one can assume that the equal means are equal to zero). We start by assuming $\left(\sigma_{P}^{2},\sigma_{Q}^{2}\right)\in {\left(1,\infty \right)}^{2}$. Let

(222) $$\begin{array}{c} \mu_{n}:=\sqrt{1+n\left(\sigma_{Q}^{2}-1\right)}, \end{array}$$

and define a sequence of quaternary real-valued random variables with probability mass functions

(223) $$Q_{n}\left(a\right):=\{\begin{array}{ll} \frac{1}{2}-\frac{1}{2n} & a=\pm 1,\\ \frac{1}{2n} & a=\pm \mu_{n}. \end{array}$$

It can be verified that, for all n∈N, $Q_{n}$ has zero mean and variance $\sigma_{Q}^{2}$. Furthermore, let

(224) $$P_{n}\left(a\right):=\{\begin{array}{ll} \frac{1}{2}-\frac{\xi }{2n} & a=\pm 1,\\ \frac{\xi }{2n} & a=\pm \mu_{n}, \end{array}$$

with

(225) $$\begin{array}{c} \xi :=\frac{\sigma_{P}^{2}-1}{\sigma_{Q}^{2}-1}. \end{array}$$

If ξ>1, for n=1,…,⌈ξ⌉, we choose $P_{n}$ arbitrarily with mean 0 and variance $\sigma_{P}^{2}$. Then,

(226) $$\begin{array}{cc} \; & \mathrm{Var}\left(P_{n}\right)=1-\frac{\xi }{n}+\frac{\xi }{n}\mu_{n}^{2}=\sigma_{P}^{2}, \end{array}$$

(227) $$\begin{array}{cc} \; & D\left(P_{n}∥Q_{n}\right)=d\left(\frac{\xi }{n}∥\frac{1}{n}\right)\to 0. \end{array}$$

Next, suppose $\min\left\{\sigma_{P}^{2},\sigma_{Q}^{2}\right\}:=\sigma^{2}<1$, then construct $P_{n}^{\prime }$ and $Q_{n}^{\prime }$ as before with variances $\frac{2\sigma_{P}^{2}}{\sigma^{2}}>1$ and $\frac{2\sigma_{Q}^{2}}{\sigma^{2}}>1$, respectively. If $P_{n}$ and $Q_{n}$ denote the random variables $P_{n}^{\prime }$ and $Q_{n}^{\prime }$ scaled by a factor of $\frac{\sigma }{\sqrt{2}}$, then their variances are $\sigma_{P}^{2}$, $\sigma_{Q}^{2}$, respectively, and $D\left(P_{n}∥Q_{n}\right)=D\left(P_{n}^{\prime }∥Q_{n}^{\prime }\right)\to 0$ as we let n→∞.

To conclude, it should be noted that the sequences of probability measures in the latter proof are defined on a four-element set. Recall that, in the earlier proof, specialized to the case of (equal means with) $\sigma_{P}^{2}\leq \sigma_{Q}^{2}$, the introduced probability measures are defined on a three-element set, and the reference probability measure *P* is fixed while referring to an equiprobable binary random variable.

### 5.4. Proof of Theorem 3

We first prove (52). Differentiating both sides of (22) gives that, for all λ∈(0,1],

(228)F′(λ)=1λχ2P∥Rλloge(229)≥1λexpD(P∥Rλ)−1loge(230)=1λexpF(λ)−1loge,

where (228) holds due to (21), (22) and (50); (229) holds by (16) and (230) is due to (21) and (50). This gives (52).

We next prove (53), and the conclusion which appears after it. In view of [16] [Theorem 8], applied to f(t):=−logt for all t>0, we get (it should be noted that, by the definition of *F* in (50), the result in [16] [(195)–(196)] is used here by swapping *P* and *Q*)

(231) $$\begin{array}{c} \lim_{\lambda \to 0^{+}}\frac{F(\lambda )}{\lambda^{2}}=\frac{1}{2}\;\chi^{2}\left(Q∥P\right)\;\log e. \end{array}$$

Since $\lim_{\lambda \to 0^{+}}F\left(\lambda \right)=0$, it follows by L’Hôpital’s rule that

(232) $$\begin{array}{c} \lim_{\lambda \to 0^{+}}\frac{F^{\prime }\left(\lambda \right)}{\lambda }=2\lim_{\lambda \to 0^{+}}\frac{F(\lambda )}{\lambda^{2}}=\chi^{2}\left(Q∥P\right)\;\log e, \end{array}$$

which gives (53). A comparison of the limit in (53) with a lower bound which follows from (52) gives

(233)limλ→0+F′(λ)λ≥limλ→0+1λ2expF(λ)−1loge(234)=limλ→0+F(λ)λ2limλ→0+expF(λ)−1F(λ)·loge(235)=limλ→0+F(λ)λ2limu→0eu−1u(236)=12χ2(Q∥P)loge,

where (236) relies on (231). Hence, the limit in (53) is twice as large as its lower bound on the right side of (236). This proves the conclusion which comes right after (53).

We finally prove the known result in (51), by showing an alternative proof which is based on (52). The function *F* is non-negative on [0,1], and it is strictly positive on (0,1] if P≠Q. Let P≠Q (otherwise, (51) is trivial). Rearranging terms in (52) and integrating both sides over the interval [λ,1], for λ∈(0,1], gives that

(237)∫λ1F′(t)expF(t)−1dt≥∫λ1dttloge(238)=log1λ,∀λ∈(0,1].

The left side of (237) satisfies

(239)∫λ1F′(t)expF(t)−1dt=∫λ1F′(t)exp−F(t)1−exp−F(t)dt(240)=∫λ1ddtlog1−exp−F(t)dt(241)=log1−exp−D(P∥Q)1−exp−F(λ),

where (241) holds since F(1)=D(P∥Q) (see (50)). Combining (237)–(241) gives

(242) $$\begin{array}{c} \frac{1-\exp\left(-D(P∥Q)\right)}{1-\exp\left(-F(\lambda )\right)}\geq \frac{1}{\lambda },\;\forall \;\lambda \in \left(0,1\right], \end{array}$$

which, due to the non-negativity of *F*, gives the right side inequality in (51) after rearrangement of terms in (242).

### 5.5. Proof of Theorem 4

**Lemma** **3.** *Let $f_{0}:\left(0,\infty \right)\to R$ be a convex function with $f_{0}\left(1\right)=0$, and let ${\left\{f_{k}\left(\cdot \right)\right\}}_{k=0}^{\infty }$ be defined as in (58). Then, ${\left\{f_{k}\left(\cdot \right)\right\}}_{k=0}^{\infty }$ is a sequence of convex functions on (0,∞), and*

(243) $$\begin{array}{c} f_{k}\left(x\right)\geq f_{k+1}\left(x\right),\;\forall \;x>0,\;\;k\in \left\{0,1,\ldots \right\}. \end{array}$$

**Proof.**  We prove the convexity of $\left\{f_{k}\left(\cdot \right)\right\}$ on (0,∞) by induction. Suppose that $f_{k}\left(\cdot \right)$ is a convex function with $f_{k}\left(1\right)=0$ for a fixed integer k≥0. The recursion in (58) yields $f_{k+1}\left(1\right)=0$ and, by the change of integration variable $s:=\left(1-x\right)s^{\prime }$,

(244) $$\begin{array}{cc} f_{k+1}\left(x\right) & =\int_{0}^{1}f_{k}\left(s^{\prime }x-s^{\prime }+1\right)\;\frac{ds^{\prime }}{s^{\prime }},\;x>0. \end{array}$$

Consequently, for t∈(0,1) and x≠y with x,y>0, applying (244) gives

(245)fk+1((1−t)x+ty)=∫01fks′[(1−t)x+ty]−s′+1ds′s′(246)=∫01fk(1−t)(s′x−s′+1)+t(s′y−s′+1)ds′s′(247)≤(1−t)∫01fk(s′x−s′+1)ds′s′+t∫01fk(s′y−s′+1)ds′s′(248)=(1−t)fk+1(x)+tfk+1(y),

where (247) holds since $f_{k}\left(\cdot \right)$ is convex on (0,∞) (by assumption). Hence, from (245)–(248), $f_{k+1}\left(\cdot \right)$ is also convex on (0,∞) with $f_{k+1}\left(1\right)=0$. By mathematical induction and our assumptions on $f_{0}$, it follows that ${\left\{f_{k}\left(\cdot \right)\right\}}_{k=0}^{\infty }$ is a sequence of convex functions on (0,∞) which vanish at 1.

We next prove (243). For all x,y>0 and k∈{0,1,…},

(249)fk+1(y)≥fk+1(x)+fk+1′(x)(y−x)(250)=fk+1(x)+fk(x)x−1(y−x),

where (249) holds since $f_{k}\left(\cdot \right)$ is convex on (0,∞), and (250) relies on the recursive equation in (58). Substituting y=1 into (249)–(250), and using the equality $f_{k+1}\left(1\right)=0$, gives (243). □

We next prove Theorem 4. From Lemma 3, it follows that $D_{f_{k}}\left(P∥Q\right)$ is an *f*-divergence for all integers k≥0, and the non-negative sequence $\{D_{f_{k}}{\left(P∥Q\right)\}}_{k=0}^{\infty }$ is monotonically non-increasing. From (21) and (58), it also follows that, for all λ∈[0,1] and integer k∈{0,1,…},

(251)Dfk+1(Rλ∥P)=∫pfk+1rλpdμ(252)=∫p∫0(p−q)λ/pfk(1−s)dssdμ(253)=∫p∫0λfk1+(q−p)s′pds′s′dμ(254)=∫0λ∫pfkrs′pdμds′s′(255)=∫0λDfk(Rs′∥P)ds′s′,

where the substitution $s:=\frac{\left(p-q\right)s^{\prime }}{p}$ is invoked in (253), and then (254) holds since $\frac{r_{s^{\prime }}}{p}=1+\frac{\left(q-p\right)\;s^{\prime }}{p}$ for $s^{\prime }\in \left[0,1\right]$ (this follows from (21)) and by interchanging the order of the integrations.

### 5.6. Proof of Corollary 5

Combining (60) and (61) yields (58); furthermore, $f_{0}:\left(0,\infty \right)\to R$, given by $f_{0}\left(x\right)=\frac{1}{x}-1$ for all x>0, is convex on (0,∞) with $f_{0}\left(1\right)=0$. Hence, Theorem 4 holds for the selected functions ${\left\{f_{k}\left(\cdot \right)\right\}}_{k=0}^{\infty }$ in (61), which therefore are all convex on (0,∞) and vanish at 1. This proves that (59) holds for all λ∈[0,1] and k∈{0,1,…}. Since $f_{0}\left(x\right)=\frac{1}{x}-1$ and $f_{1}\left(x\right)=-\log_{e}\left(x\right)$ for all x>0 (see (60) and (61)), then, for every pair of probability measures *P* and *Q*:

(256) $$\begin{array}{c} D_{f_{0}}\left(P∥Q\right)=\chi^{2}\left(Q∥P\right),\;D_{f_{1}}\left(P∥Q\right)=\frac{1}{\log e}\;D\left(Q∥P\right). \end{array}$$

Finally, combining (59), for k=0, together with (256), gives (22) as a special case.

### 5.7. Proof of Theorem 5 and Corollary 6

For an arbitrary measurable set E⊆X, we have from (62)

(257) $$\begin{array}{c} \mu_{C}\left(E\right)=\int_{E}\frac{1_{C}\left(x\right)}{\mu (C)}\;d\mu \left(x\right), \end{array}$$

where $1_{C}:X\to \left\{0,1\right\}$ is the indicator function of C⊆X, i.e., $1_{C}\left(x\right):=1\left\{x\in C\right\}$ for x∈X. Hence,

(258) $$\begin{array}{c} \frac{d\mu_{C}}{d\mu }\;\left(x\right)=\frac{1_{C}\left(x\right)}{\mu (C)},\;\forall \;x\in X, \end{array}$$

and

(259)D(μC∥μ)=∫XfdμCdμdμ(260)=∫Cf1μ(C)dμ(x)+∫X\Cf(0)dμ(x)(261)=μ(C)f1μ(C)+μ(X\C)f(0)(262)=f˜μ(C)+(1−μ(C))f(0),

where the last equality holds by the definition of $\tilde{f}$ in (63). This proves Theorem 5. Corollary 6 is next proved by first proving (67) for the Rényi divergence. For all α∈(0,1)∪(1,∞),

(263)DαμC∥μ=1α−1log∫XdμCdμαdμ(264)=1α−1log∫C1μ(C)αdμ(265)=1α−1log1μ(C)αμ(C)(266)=log1μ(C).

The justification of (67) for α=1 is due to the continuous extension of the order-α Rényi divergence at α=1, which gives the relative entropy (see (13)). Equality (65) is obtained from (67) at α=1. Finally, (66) is obtained by combining (15) and (67) with α=2.

### 5.8. Proof of Theorem 6

Equation (100) is an equivalent form of (27). From (91) and (100), for all α∈[0,1],

(267)1logeSα(P∥Q)=α1logeKα(P∥Q)+(1−α)1logeK1−α(Q∥P)(268)=α∫0αsDϕs(P∥Q)ds+(1−α)∫01−αsDϕs(Q∥P)ds(269)=α∫0αsDϕs(P∥Q)ds+(1−α)∫α1(1−s)Dϕ1−s(Q∥P)ds.

Regarding the integrand of the second term in (269), in view of (18), for all s∈(0,1)

(270)Dϕ1−s(Q∥P)=1(1−s)2·χ2Q∥(1−s)P+sQ(271)=1s2·χ2P∥(1−s)P+sQ(272)=Dϕs(P∥Q),

where (271) readily follows from (9). Since we also have $D_{\phi_{1}}\left(P∥Q\right)=\chi^{2}\left(P∥Q\right)=D_{\phi_{0}}\left(Q∥P\right)$ (see (18)), it follows that

(273) $$\begin{array}{c} D_{\phi_{1-s}}\left(Q∥P\right)=D_{\phi_{s}}\left(P∥Q\right),\;s\in \left[0,1\right]. \end{array}$$

By using this identity, we get from (269) that, for all α∈[0,1]

(274)1logeSα(P∥Q)=α∫0αsDϕs(P∥Q)ds+(1−α)∫α1(1−s)Dϕs(P∥Q)ds(275)=∫01gα(s)Dϕs(P∥Q)ds,

where the function $g_{\alpha }:\left[0,1\right]\to R$ is defined in (102). This proves the integral identity (101).

The lower bounds in (103) and (104) hold since, if f:(0,∞)→R is convex, continuously twice differentiable and strictly convex at 1, then

(276) $$\begin{array}{c} \mu_{\chi^{2}}\left(Q_{X},W_{Y|X}\right)\leq \mu_{f}\left(Q_{X},W_{Y|X}\right), \end{array}$$

(see, e.g., [46] [Proposition II.6.5] and [50] [Theorem 2]). Hence, this holds in particular for the *f*-divergences in (95) and (96) (since the required properties are satisfied by the parametric functions in (97) and (98), respectively). We next prove the upper bound on the contraction coefficients in (103) and (104) by relying on (100) and (101), respectively. In the setting of Definition 7, if $P_{X}\neq Q_{X}$, then it follows from (100) that for α∈(0,1],

(277)Kα(PY∥QY)Kα(PX∥QX)=∫0αsDϕs(PY∥QY)ds∫0αsDϕs(PX∥QX)ds(278)≤∫0αsμϕs(QX,WY|X)Dϕs(PX∥QX)ds∫0αsDϕs(PX∥QX)ds(279)≤sups∈(0,α]μϕs(QX,WY|X).

Finally, taking the supremum of the left-hand side of (277) over all probability measures $P_{X}$ such that $0<K_{\alpha }\left(P_{X}∥Q_{X}\right)<\infty$ gives the upper bound on $\mu_{k_{\alpha }}\left(Q_{X},W_{Y|X}\right)$ in (103). The proof of the upper bound on $\mu_{s_{\alpha }}\left(Q_{X},W_{Y|X}\right)$, for all α∈[0,1], follows similarly from (101), since the function $g_{\alpha }\left(\cdot \right)$ as defined in (102) is positive over the interval (0,1).

### 5.9. Proof of Corollary 7

The upper bounds in (106) and (107) rely on those in (103) and (104), respectively, by showing that

(280) $$\begin{array}{c} \underset{s\in (0,1]}{\sup}\mu_{\phi_{s}}\left(Q_{X},W_{Y|X}\right)\leq \mu_{\chi^{2}}\left(W_{Y|X}\right). \end{array}$$

Inequality (280) is obtained as follows, similarly to the concept of the proof of [51] [Remark 3.8]. For all s∈(0,1] and $P_{X}\neq Q_{X}$,

Dϕs(PXWY|X∥QXWY|X)Dϕs(PX∥QX)(281)=χ2(PXWY|X∥(1−s)PXWY|X+sQXWY|X)χ2(PX∥(1−s)PX+sQX)(282)≤μχ2((1−s)PX+sQX,WY|X)(283)≤μχ2(WY|X),

where (281) holds due to (18), and (283) is due to the definition in (105).

### 5.10. Proof of Proposition 3

The lower bound on the contraction coefficients in (108) and (109) is due to (276). The derivation of the upper bounds relies on [49] [Theorem 2.2], which states the following. Let f:[0,∞)→R be a three–times differentiable, convex function with f(1)=0, $f^{\prime \prime }\left(1\right)>0$, and let the function z:(0,∞)→R defined as $z\left(t\right):=\frac{f(t)-f(0)}{t}$, for all t>0, be concave. Then,

(284) $$\begin{array}{c} \mu_{f}\left(Q_{X},W_{Y|X}\right)\leq \frac{f^{\prime }\left(1\right)+f\left(0\right)}{f^{\prime \prime }\left(1\right)\;Q_{\min}}\cdot \mu_{\chi^{2}}\left(Q_{X},W_{Y|X}\right). \end{array}$$

For α∈(0,1], let $z_{\alpha ,1}:\left(0,\infty \right)\to R$ and $z_{\alpha ,2}:\left(0,\infty \right)\to R$ be given by

(285) $$\begin{array}{cc} z_{\alpha ,1}\left(t\right) & :=\frac{k_{\alpha }\left(t\right)-k_{\alpha }\left(0\right)}{t},\;t>0, \end{array}$$

(286) $$\begin{array}{cc} z_{\alpha ,2}\left(t\right) & :=\frac{s_{\alpha }\left(t\right)-s_{\alpha }\left(0\right)}{t},\;t>0, \end{array}$$

with $k_{\alpha }$ and $s_{\alpha }$ in (97) and (98). Straightforward calculus shows that, for α∈(0,1] and t>0,

(287)1logezα,1″(t)=−α2+2α(1−α)tt2α+(1−α)t2<0,1logezα,2″(t)=−α2α+2(1−α)tt2α+(1−α)t2(288)−2(1−α)t3loge1+(1−α)tα−(1−α)tα+(1−α)t−(1−α)2t22α+(1−α)t2.

The first term on the right side of (288) is negative. For showing that the second term is also negative, we rely on the power series expansion $\log_{e}\left(1+u\right)=u-\frac{1}{2}u^{2}+\frac{1}{3}u^{3}-\ldots$ for u∈(−1,1]. Setting $u:=-\frac{x}{1+x}$, for x>0, and using Leibnitz theorem for alternating series yields

(289) $$\begin{array}{c} \log_{e}\left(1+x\right)=-\log_{e}\left(1-\frac{x}{1+x}\right)>\frac{x}{1+x}+\frac{x^{2}}{2{\left(1+x\right)}^{2}},\;x>0. \end{array}$$

Consequently, setting $x:=\frac{(1-\alpha )t}{\alpha }\in \left[0,\infty \right)$ in (289), for t>0 and α∈(0,1], proves that the second term on the right side of (288) is negative. Hence, $z_{\alpha ,1}^{\prime \prime }\left(t\right),\;z_{\alpha ,2}^{\prime \prime }\left(t\right)<0$, so both $z_{\alpha ,1},z_{\alpha ,2}:\left(0,\infty \right)\to R$ are concave functions.

In view of the satisfiability of the conditions of [49] [Theorem 2.2] for the *f*-divergences with $f=k_{\alpha }$ or $f=s_{\alpha }$, the upper bounds in (108) and (109) follow from (284), and also since

(290)kα(0)=0,kα′(1)=αloge,kα″(1)=α2loge,(291)sα(0)=−(1−α)logα,sα′(1)=(2α−1)loge,sα″(1)=(1−3α+3α2)loge.

### 5.11. Proof of Proposition 4

In view of (24), we get

(292)D(PY∥QY)D(PX∥QX)=∫01χ2(PY∥(1−s)PY+sQY)dss∫01χ2(PX∥(1−s)PX+sQX)dss(293)≤∫01μχ2((1−s)PX+sQX,WY|X)χ2(PX∥(1−s)PX+sQX)dss∫01χ2(PX∥(1−s)PX+sQX)dss(294)≤sups∈[0,1]μχ2((1−s)PX+sQX,WY|X).

In view of (119), the distributions of $X_{s}$ and $Y_{s}$, and since $\left(\left(1-s\right)P_{X}+sQ_{X}\right)W_{Y|X}=\left(1-s\right)P_{Y}+sQ_{Y}$ holds for all s∈[0,1], it follows that

(295) $$\begin{array}{c} \rho_{m}\left(X_{s};Y_{s}\right)=\sqrt{\mu_{\chi^{2}}\left(\left(1-s\right)P_{X}+sQ_{X},\;W_{Y|X}\right)},\;s\in \left[0,1\right], \end{array}$$

which, from (292)–(295), implies that

(296) $$\begin{array}{c} \underset{s\in [0,1]}{\sup}\rho_{m}\left(X_{s};Y_{s}\right)\geq \sqrt{\frac{D(P_{Y}∥Q_{Y})}{D(P_{X}∥Q_{X})}}. \end{array}$$

Switching $P_{X}$ and $Q_{X}$ in (292)–(294) and using the mapping s↦1−s in (294) gives (due to the symmetry of the maximal correlation)

(297) $$\begin{array}{c} \underset{s\in [0,1]}{\sup}\rho_{m}\left(X_{s};Y_{s}\right)\geq \sqrt{\frac{D(Q_{Y}∥P_{Y})}{D(Q_{X}∥P_{X})}}, \end{array}$$

and, finally, taking the maximal lower bound among those in (296) and (297) gives (120).
